# The SVP–FRO6 Module Integrates the Control of Leaf Senescence and Flowering in *Arabidopsis*

**DOI:** 10.1111/pce.70677

**Published:** 2026-06-18

**Authors:** Hyunsu Park, Yeong Seon Yoon, Sanghoon Park, Ukcheol Jeong, Hyeyoung Choi, Hyunwoo Oh, Dovran Shamamedov, Zhonghai Li, Hye Ryun Woo

**Affiliations:** ^1^ Department of New Biology Daegu Gyeongbuk Institute of Science and Technology (DGIST) Daegu Korea; ^2^ State Key Laboratory of Tree Genetics and Breeding, College of Biological Sciences and Technology Beijing Forestry University Beijing China; ^3^ New Biology Research Center Daegu Gyeongbuk Institute of Science and Technology (DGIST) Daegu Korea

**Keywords:** Fe accumulation, flowering time, FRO6, leaf senescence, SVP

## Abstract

Plants coordinate major developmental transitions by integrating intrinsic genetic programs with environmental signals, where nutrient availability serves as a critical external factor shaping growth and fitness. Iron (Fe), an essential micronutrient for cellular respiration and photosynthesis, is strictly regulated through sophisticated uptake and transport systems, yet its specific role in timing developmental transitions, particularly leaf senescence and flowering, remains largely unresolved. Here, we show that total Fe concentration gradually increases with leaf age and excessive Fe accumulation accelerates leaf senescence in *Arabidopsis*. We identify Ferric Reduction Oxidase 6 (FRO6), a ferric reductase family protein, as a novel negative regulator of leaf senescence that modulates Fe accumulation in leaves. Our results indicate that FRO6 contributes to the regulation of leaf senescence across a broad range of Fe availability, from deficiency to excess. Furthermore, we find that FRO6 regulates the transcript levels of *MYC2*, a master regulator of jasmonic acid signalling and environmental stress responses, to modulate leaf senescence. In addition, Short Vegetative Phase (SVP), a key floral repressor, directly activates *FRO6* expression by binding to its promoter, thereby establishing an SVP–FRO6 regulatory module that coordinates the timing of leaf senescence and floral transition. Collectively, these results provide new mechanistic insights into how Fe homoeostasis in leaves interfaces with leaf senescence and reproductive development, highlighting Fe as a pivotal regulator of plant developmental timing.

## Summary statement

Here, we show that total iron (Fe) concentration gradually increases with leaf age and excessive Fe accumulation accelerates leaf senescence in *Arabidopsis*. We identify Ferric Reduction Oxidase 6 as a negative regulator of leaf senescence that modulates Fe accumulation in leaves.

## 1 Introduction

Plants finely orchestrate key developmental transitions through the integration of endogenous developmental programs and environmental cues to optimize fitness throughout their life cycle (Lim et al. 2007; X. Li et al. 2025). As the primary photosynthetic organ, the leaf undergoes a series of distinct developmental phases—initiation, growth, maturation and ultimately, senescence (Bar and Ori 2014). Leaf senescence is a genetically programmed and tightly regulated developmental process that is primarily determined by leaf age, yet highly plastic in response to internal and external signals (Guo 2013; J. Kim, Kim, Lyu, et al. 2018; Woo et al. 2019). Its onset and progression are modulated by phytohormones, including abscisic acid (ABA), ethylene, and jasmonic acid (JA), as well as by diverse environmental stresses, such as nutrient imbalance, prolonged darkness and drought (Guo et al. 2021; Tan et al. 2023). At the cellular level, senescing leaves exhibit a progressive decline in anabolic processes, such as photosynthesis and amino acid biosynthesis, accompanied by enhanced catabolic activities, including chlorophyll and starch degradation (Nam 1997; Mayta et al. 2019). This controlled dismantling of cellular components enables the remobilization of nutrients to developing sink organs, thereby optimizing resource reallocation and enhancing reproductive success in response to fluctuating developmental programs and environmental stimuli (Woo et al. 2013; Q. Chen et al. 2019).

Leaf senescence is governed by multilayered regulatory mechanisms, including chromatin remodelling as well as transcriptional, post‐transcriptional, translational and post‐translational control (Woo et al. 2016; U. Jeong et al. 2025). Extensive transcriptional reprogramming underlies leaf senescence, with senescence‐regulatory transcription factors (TFs) modulating the expression of thousands of senescence‐associated genes (*SAGs*) (Guo and Gan 2005; Z. Li et al. 2018). Prominent TF families include the NAM/ATAF/CUC (NAC) proteins, such as ACTIVATING FACTOR 1 (ATAF1) and ORESARA 1 (ORE1), which act as positive regulators of leaf senescence, and JUNGBRUNNEN 1 (JUB1) and VND‐INTERACTING 1 (VNI1), which function as negative regulators (J. H. Kim et al. 2018; Y.‐M. Zhang, Guo, et al. 2021; S. Li et al. 2024). Similarly, members of the WRKY TF family, including WRKY47, WRKY55, and WRKY75, also play key roles in modulating leaf senescence (Y. Wang, Cui, et al. 2020; H. Zhang, Zhang, et al. 2021; X. Cui et al. 2024). Despite these advances, our understanding of how plants dynamically integrate developmental signals with environmental and nutritional cues to coordinate the complex regulatory networks governing leaf senescence is still incomplete.

The shift from vegetative to reproductive growth represents another key developmental transition controlled by multiple signalling pathways, including the autonomous, vernalization, and gibberellin (GA) pathways, alongside environmental cues, such as photoperiod, temperature fluctuations, water availability, and nutrient status (Niu et al. 2024; Cho et al. 2025). Central floral integrators such as FLOWERING LOCUS T (FT) and SUPPRESSOR OF OVEREXPRESSION OF CONSTANS 1 (SOC1) promote floral induction by activating floral meristem identity genes, like *APETALA1* (*AP1*) and *LEAFY* (*LFY*) (Rehman et al. 2023). In contrast, the MADS‐box TFs FLOWERING LOCUS C (FLC) and Short Vegetative Phase (SVP) repress flowering by directly binding CArG motifs within the regulatory regions of *FT* and *SOC1* (J. Lee and Lee 2010; Gregis et al. 2013; J. H. Lee et al. 2017). Beyond its role as a floral repressor, SVP functions as an integrative hub that links developmental timing with environmental signals by modulating GA biosynthesis, ABA‐mediated drought tolerance, and cold stress responses (Seo et al. 2009; Andrés et al. 2014; Z. Wang et al. 2018). Together, these regulatory components form a finely tuned network ensuring that flowering occurs at an appropriate developmental stage and under favourable environmental conditions.

Accumulating evidence indicates that flowering time and leaf senescence are often coordinately regulated. Circadian clock components such as EARLY FLOWERING 4 (ELF4), GIGANTEA (GI), and PSEUDO‐RESPONSE REGULATOR 9 (PRR9) participate in both processes (H. Kim, Kim, Vu et al. 2018; Y. Zhang et al. 2018). For example, ELF4 suppresses GI‐mediated activation of *ORE1* by inhibiting GI binding to the *ORE1* promoter, whereas PRR9 promotes leaf senescence by directly activating *ORE1* expression while repressing *miRNA164*, a post‐transcriptional repressor of *ORE1* (Y. Kim et al. 2012; H. Kim et al. 2020). Additionally, WRKY1 promotes flowering by repressing *FLC* while simultaneously facilitating nitrogen recycling during leaf senescence (W. Zhang et al. 2024). However, whether key floral regulators, such as SVP, participate in the control of leaf senescence has remained largely unexplored.

Iron (Fe) is an essential micronutrient required for a wide range of biological processes, acting as a cofactor for metalloproteins involved in cellular respiration and photosynthesis (Kobayashi et al. 2019; Herlihy et al. 2020). Fe deficiency severely impairs mitochondrial respiration by disrupting heme biosynthesis and Fe–sulphur (S) cluster assembly (Bashir et al. 2011; Jain and Connolly 2013). Insufficient Fe causes leaf chlorosis by hindering chlorophyll biosynthesis and the assimilation of nitrogen (N) and S (J. Li et al. 2021; Khan et al. 2022; Ni et al. 2022). Beyond its central role in primary metabolism, Fe homoeostasis has been reported to be associated with leaf senescence. Fe levels increase with leaf age under supraoptimal Fe supply in *Brassica napus* (Sági‐Kazár et al. 2021), and *Arabidopsis* rosette leaves exhibit differential Fe‐deficiency responses depending on reproductive developmental stages (Ngigi et al. 2025). Moreover, loss of the Fe‐storage protein FERRITIN 1 (FER1) leads to premature leaf senescence in *Arabidopsis* (Murgia et al. 2007), and the H2B histone variant HTB4 delays leaf senescence by modulating Fe homoeostasis (Yang et al. 2023). Fe status also influences flowering time; Fe deficiency lengthens circadian period and delays flowering through Fe‐responsive basic helix–loop–helix (bHLH) TFs (bHLH38, bHLH100 and bHLH101) that interact with CONSTANS (CO) to repress *FT* expression (Y. Y. Chen et al. 2013; W. Chen et al. 2021).

Although Fe is abundant in soil, it is mostly present as insoluble ferric iron (Fe^3+^) and must be reduced to the ferrous form (Fe^2+^) for uptake (Ning et al. 2023). In *Arabidopsis*, Fe acquisition involves: (i) rhizosphere acidification by the plasma membrane H^+^‐ATPase 2 (AHA2), (ii) reduction of Fe^3+^ to Fe^2+^ by the ferric‐chelate reductase Ferric Reduction Oxidase 2 (FRO2) and (iii) subsequent uptake of Fe^2+^ via iron‐regulated transporter 1 (IRT1) (Rajniak et al. 2018; Liang 2022). The *Arabidopsis FRO* gene family comprises eight members with distinct subcellular localization patterns: FRO2, FRO4, FRO5 and FRO6 localize to the plasma membrane; FRO3 and FRO8 are predicted to be mitochondrial membrane; and FRO7 localizes to the chloroplast membrane (Jain et al. 2014). Functional studies have revealed that FRO4 and FRO5 redundantly mediate copper reduction at the root surface (Bernal et al. 2012), while FRO7 is essential for chloroplast ferric reductase activity (J. Jeong et al. 2008). Furthermore, knockdown of the vacuolar membrane‐localized OsFRO1 alleviates excess Fe‐induced reactive oxygen species (ROS) accumulation (L. Li et al. 2019). Nevertheless, the functional significance of FRO‐dependent Fe homoeostasis in coordinating major developmental processes, particularly leaf senescence and floral transition, has yet to be elucidated.

In this study, we demonstrated that excess Fe accumulation–induced premature leaf senescence in *Arabidopsis* and identified FRO6 as a previously uncharacterized negative regulator of leaf senescence that modulates Fe accumulation in leaves. Transcriptomic analysis revealed that loss of *FRO6* function altered JA‐associated pathways, and genetic analysis placed MYC2 downstream of FRO6 in the regulation of leaf senescence. We further showed that the floral repressor SVP directly activated *FRO6* transcription, thereby establishing an SVP–FRO6 regulatory axis that coordinates the timing of leaf senescence and floral transition. Collectively, our findings uncover a mechanistic framework that links Fe homoeostasis to major developmental transitions, providing new insights into how plants couple nutrient status with leaf senescence and reproductive timing.

## 2 Methods

### 2.1 Plant Materials and Growth Conditions

*Arabidopsis thaliana* Columbia (Col) ecotype was used as the wild‐type reference. T‐DNA insertion mutants of *FRO6*, including *fro6‐1* (SALK_099597; insertion in the eighth exon), *fro6‐2* (CS802040; −192 bp from the translation start site), and *fro6‐3Dominant* (*fro6‐3D*) (SALK_138745; −3831 bp from the translation start site)—along with *myc2* (SALK_017005; insertion in the first exon) and *svp* (SALK_066466; insertion in the second intron) were obtained from public seed stocks and verified by polymerase chain reaction (PCR)‐based genotyping. Double mutants (*fro6‐1 myc2* and *fro6‐1 svp*) were generated by genetic crossing and confirmed by PCR.

For overexpression constructs, the coding sequences of *FRO6* and *SVP* were amplified via reverse transcription (RT)‐PCR, cloned into pENTR/D‐TOPO (Invitrogen, USA), and recombined into the Gateway‐compatible *pCsVMV* binary vector containing a C‐terminal Green Fluorescence Protein (GFP) tag using Gateway LR clonase Ⅱ enzyme mix (Invitrogen, USA). The resulting constructs were introduced to Col or *fro6‐1* plants via *Agrobacterium tumefaciens*–mediated floral dip transformation to generate *FRO6‐OE*, *SVP‐OE* and *SVP‐OE*/*fro6‐1* lines.

To generate *fro6‐c1* and *fro6‐c2* mutants using the CRISPR–Cas9 editing system, two pairs of guide RNAs targeting the middle (gRNA1/2) and terminal (gRNA3/4) regions of *FRO6* were designed using CCTop (https://cctop.cos.uni-heidelberg.de/). The gRNA expression cassettes were amplified using pEN‐2xAtU6 as a template and cloned into the pKIR1.1 vector as previously described (Tsutsui and Higashiyama 2017; Decaestecker et al. 2019). Transformed lines were selected based on Red Fluorescence Protein (RFP) signal in the seed coat and validated by Sanger sequencing. To eliminate the transgene and minimize potential off‐target effects, RFP‐negative seeds were selected for subsequent phenotypic analysis. All primers used for genotyping and vector construction are listed in Table S1. Plants were grown at 22°C under long‐day (LD) conditions (16‐h light/8‐h dark) in a controlled growth room.

### 2.2 Determination of Fe Content

Fe content was quantified as described by Gautam et al. (2021) with minor modifications. Dried leaf tissue (5 mg) was digested in 65% nitric acid at 95°C for 6 h until no visible precipitate remained. After cooling, 30% hydrogen peroxide was added, and samples were incubated for an additional 2 h to obtain a clear digest. Samples and Fe standards were then reacted with the bathophenanthroline disulfonate (BPDS) assay solution (1 mM BPDS, 0.6 M sodium acetate, 0.48 M hydroxylamine hydrochloride). Absorbance was measured at 535 nm using a Synergy HTX Multi‐Mode Reader (BioTek, USA). Fe concentrations were determined from a standard curve and normalized to dry weight.

### 2.3 RNA Extraction and Quantitative Gene Expression Analysis

Total RNA was extracted using QIAzol (QIAGEN, Germany), followed by RT with the PrimeScript RT reagent kit (TAKARA Bio, Japan). Quantitative PCR (qPCR) was carried out using the TOPreal SYBR Green qPCR Premix (Enzynomics, Republic of Korea) on a CFX96 Touch Real‐Time PCR detection system (Bio‐Rad, USA). Relative transcript levels were quantified using the comparative threshold (ΔΔCt) method, and normalized to *glyceraldehyde‐3‐phosphate dehydrogenase C2* (*GAPC2*; *AT1G13440*) or *Protein Phosphatase 2A* (*PP2A*; *AT1G13320*). All gene‐specific primers used for gene expression analyses are listed in Table S1.

### 2.4 Leaf Senescence Assays

All leaf senescence assays were conducted using the third and fourth rosette leaves. Age‐dependent senescence was assessed with leaves harvested from plants at 6‐day intervals between 12 and 30 days after emergence (DAE). For the dark‐induced senescence assay, leaves detached at 12 DAE were floated on 3 mM 2‐(*N*‐morpholino)ethanesulfonic acid (MES) buffer (pH 5.8) and incubated in complete darkness at 22°C for the indicated durations. For excess Fe‐induced senescence, 12‐DAE leaves were floated on 3 mM MES buffer supplemented with 100–300 µM ferric sulphate (iron[III] sulphate hydrate; Sigma‐Aldrich, USA) and incubated under LD conditions; MES buffer without Fe supplementation was the mock control.

The low‐Fe (LF) assay was performed as described by J. Lee, Kang, et al. (2025) with minor modifications. Surface‐sterilized seeds were stratified at 4°C in the dark for 2 days and then sown on half‐strength Murashige and Skoog (MS) medium containing 1% (w/v) sucrose and 0.8% (w/v) agar (pH 5.7). The sufficient‐Fe (SF) solution contained 5 mM KNO_3_, 2 mM MgSO_4_, 1 mM CaCl_2_, 0.1 mM Fe‐ethylenediaminetetraacetate (Fe‐EDTA), 50 μM H_3_BO_3_, 12 μM MnSO_4_, 1 μM ZnCl_2_, 1 μM CuSO_4_, 0.2 μM Na_2_MoO_4_, and 10 mM potassium phosphate (pH 5.7). For the LF solution, 0.1 mM Fe‐EDTA was reduced to 0.5 μM. For senescence phenotype analysis, seedlings at 5‐day‐after‐germination (DAG) were transferred from half‐strength MS to SF or LF medium and grown for an additional 18 days. Alternatively, 5‐DAG seedlings were transferred to soil (vermiculite:peat moss, 1:9) with or without 1 g L^−1^ CaCO_3_ (Rodríguez‐Celma et al. 2016) to induce alkaline‐based Fe deficiency and then grown for 4 weeks. Pots were submerged weekly for 5 h in either LF (for CaCO_3_‐treated soil) or SF solution (for untreated soil).

Leaf senescence progression was evaluated by measuring photochemical efficiency (*F*_v_/*F*_m_), electron transfer rate (ETR), and maximum ETR (ETR_max_), as well as by quantifying senescence–associated marker gene expression via RT‐qPCR, as previously described (Woo et al. 2001; J. H. Kim et al. 2018; Park et al. 2022). Rapid light response curves (RLCs) were generated by stepwise increase in actinic light intensity (levels 1–20), and RLC‐based ETR values were recorded at each step to calculate ETR_max_.

### 2.5 mRNA‐Sequencing (mRNA‐Seq) and Differential Expression Analysis

For mRNA‐seq analysis, total RNA was extracted from three independent biological replicates representing two experimental setups: (i) third and fourth rosette leaves of Col plants detached at 12 DAE and treated with or without 200 µM ferric sulphate for 1 day under LD conditions, and (ii) third and fourth rosette leaves of Col and *fro6‐1* plants harvested at 24 DAE. Libraries were constructed using the TruSeq Stranded mRNA Library Preparation Kit (Illumina, USA) and sequenced on a NovaSeq X plus system (Illumina, USA). Sequenced data were processed through the GALAXY web platform (Fallmann et al. 2019). Adapter trimming was performed with Cutadapt (v5.2), trimmed reads were aligned to the TAIR10 reference genome using BWA‐MEM2 (v2.3), and gene‐level counts were generated using featureCounts (v2.1.1). Differential expression analysis was performed using DESeq. 2 (v2.11.40.2). Genes with an adjusted *p* < 0.05 and an absolute log_2_ fold change (|log_2_ FC|) > 0.8 were considered differentially expressed. Gene Ontology (GO) enrichment analysis was performed using the DAVID Functional Annotation Tool (https://david.ncifcrf.gov/) with TAIR10 as the background reference.

### 2.6 Comparative Transcriptomic Analyses

To compare transcriptomic changes between age‐dependent and excess Fe‐induced leaf senescence, age‐dependent DEGs were defined as those exhibiting *|*log_2_(FC)| > 1.5 across different leaf ages (12–30 DAE; Woo et al. 2016), whereas Fe‐responsive DEGs were defined as described above. Overlapping DEGs were identified using Venn diagram analysis (Venny 2.1.0; Oliveros 2007).

Expression patterns of 53 Fe transport‐ and/or Fe homoeostasis–associated genes annotated in TAIR10 as encoding Fe transporters, homoeostasis regulators, or ferric–ferrous redox‐related proteins were analyzed across three independent transcriptome data sets: age‐dependent senescence (Woo et al. 2016), dark‐induced senescence (0–3 days after treatment [DAT]; H. Kim, Kim, Vu, et al. 2018), and excess Fe‐induced senescence (mock vs. 200 µM ferric sulphate treatment; Supporting Information S1). Genes with |log_2_(FC)| > 0.8 were classified as DEGs, and transcript levels are provided in Table S2.

To compare the excess Fe‐induced and *fro6‐1*‐mediated transcriptome data sets, Fe‐induced DEGs were defined as described above, and 3085 *fro6‐1*–mediated DEGs (|log_2_ FC| > 0.8 with *p* < 0.05; Supporting Information S2) were identified from Col and *fro6‐1* mutant leaves. Overlapping DEGs were determined using Venn diagram analysis (Venny 2.1.0; Oliveros 2007), followed by GO enrichment analysis. For heatmap visualization, all overlapping DEGs with *p* < 0.05 were included, irrespective of FC magnitude. Heatmaps of pathways of interest were generated using the R package pheatmap (v1.0.12; https://CRAN.R-project.org/package=pheatmap/).

### 2.7 Histochemical Analysis

To generate the *pFRO6::β‐Glucuronidase* (*GUS*) reporter line, a 1‐kb promoter region of *FRO6* was amplified, cloned into pENTR/D‐TOPO (Invitrogen, USA), and recombined into the *pKGWFS7* destination vector (Karimi et al. 2002). Transgenic plants were generated by floral dip transformation. GUS staining was performed on 2‐week‐old seedlings and various organs from 5 to 6‐week‐old plants following standard protocol (X. Li 2011).

### 2.8 In Silico Analysis of DNA Affinity Purification Sequencing (DAP‐seq) Data

DAP‐seq data set from O'Malley et al. (2016) was analyzed to identify TFs binding the *FRO6* promoter. Enrichment signals within the 1‐kb promoter region were extracted from narrowPeak files, and TFs with gene names and normalized enrichment signals > 80 were selected for further analysis (Table S3).

### 2.9 Transient Luciferase Assay

The reporter construct was generated by recombining the entry clone containing the 1‐kb *FRO6* promoter into the Gateway‐compatible *pGreenII 0800‐LUC* (*gGreenII 0800‐LUC*) vector, which carries a *CaMV35S* promoter‐driven *Renilla Luciferase* (REN) cassette and a promoterless *Firefly Luciferase* (LUC) cassette (Hellens et al. 2005). For the effector construct, the *gCsVMV::SVP‐eGFP* expression vector was employed. Reporter and effector plasmids were co‐transfected into *Arabidopsis* mesophyll protoplasts using a previously described polyethylene glycol–mediated transfection procedure (Wu et al. 2009). Luciferase activity was measured after 16 h using the Dual‐Luciferase Reporter Assay System (Promega, USA). LUC activity was normalized to REN activity, and results are presented as the LUC/REN ratio relative to the empty vector control.

### 2.10 Chromatin Immunoprecipitation (ChIP) Assay

ChIP was performed essentially as described by Saleh et al. (2008) with minor modifications. To generate transgenic *Arabidopsis* plants overexpressing *SVP‐MYC* (*35S::SVP‐MYC*), the *SVP* entry clone was subcloned into the *pCsVMV* binary vector under the control of the *CsVMV* promoter with a C‐terminal MYC tag, and the resulting construct was introduced into Col plants. Approximately 4 g of rosette leaves from *35S::SVP‐MYC* plants were vacuum‐infiltrated with 1% formaldehyde for 10 min, quenched with 0.1 M glycine for 5 min, rinsed with sterile water, and ground in liquid nitrogen. Nuclei were isolated, and chromatin was sonicated to obtain fragmented DNA. A 100‐µL aliquot of chromatin supernatant was diluted to 1 mL, precleared with 25 µL of Protein A/G magnetic beads (Pierce, USA) for 1 h at 4°C, and divided into two aliquots. Each aliquot was incubated overnight at 4°C with either anti‐MYC monoclonal antibody (#2276; Cell Signalling, USA) or anti‐HA monoclonal antibody (3F10; Roche, Switzerland) as a negative control. Immunoprecipitated DNA and input controls were analyzed by qPCR as described by J. H. Lee et al. (2017). *PP2A* (*AT1G69960*) served as the internal reference. All primers used for ChIP‐qPCR are listed in Table S1.

### 2.11 Flowering Time Measurement

Plants were grown in soil under LD conditions at 22°C. Flowering times were determined by recording the number of days to the first flower opening and by counting the total number of rosette leaves at bolting. Bolting was defined as the emergence of the inflorescence from the rosette centre.

### 2.12 Statistical Analysis

For statistical analysis, GraphPad Prism 10 was used. For all graphs, data were represented as the standard error of the mean. All details of the statistics applied are provided in the figure legends.

## 3 Results

### 3.1 Fe Accumulation Induces Premature Leaf Senescence in *Arabidopsis*

**Figure 1:**
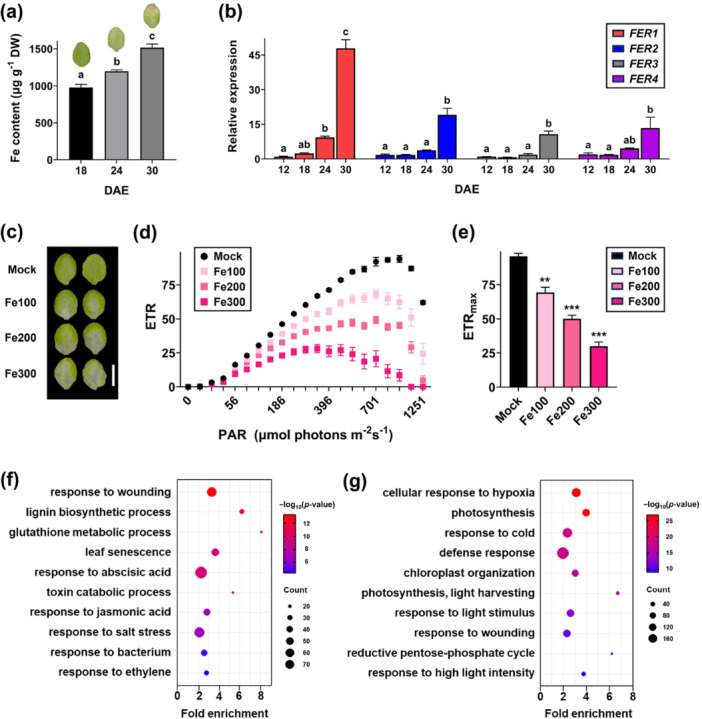
Premature leaf senescence induced by excess Fe in *Arabidopsis*. (a) Age‐dependent increase in iron (Fe) content in leaves. Total Fe content was measured in the third and fourth rosette leaves of Col plants at 18, 24, and 30 days after emergence (DAE). Representative leaf images corresponding to each sampling point are shown. Data are presented as mean ± standard error (SE; *n* = 8) and are representative of three independent experiments. (b) Upregulation of the *FERRITIN* (*FER*) genes during age‐dependent leaf senescence. Transcript levels of *FER1* to *FER4* were determined in Col leaves at 12, 18, 24, and 30 DAE, normalized to *glyceraldehyde‐3‐phosphate dehydrogenase C2* (*GAPC2*), with expression levels calculated relative to the 12‐DAE. Data are presented as mean ± SE of four independent biological replicates. (c–e) Accelerated leaf senescence phenotypes by excess Fe treatment. Representative images (c), electron transport rate (ETR) curves (d), and maximum ETR (ETR_max_) values (e) of the third and fourth rosette leaves of Col plants after 2 days of Fe treatment are shown. Treatments included 100, 200, and 300 µM ferric sulphate (Fe100, Fe200 and Fe300), as well as a mock control. Data are presented as mean ± SE (*n* = 10) and are representative of three independent experiments. Scale bar, 1 cm in (c). (f, g) Functional clustering of differentially expressed genes (DEGs) induced by excess Fe. Gene Ontology (GO) enrichment analysis was performed using DEGs identified between 200 µM ferric sulphate‐ and mock‐treated Col leaves following a 1‐day treatment. DEGs were defined using a threshold of |log_2_(fold‐change [FC])| > 0.8, with *p* < 0.05. The top 10 enriched biological process clusters and their representative terms are shown for upregulated (f) and downregulated (g) DEGs. Dot size indicates the number of intersection genes, and dot colour intensity reflects the −log_10_‐transformed adjusted *p* value (−log_10_[*p* value]). Statistical significance in (a, b) was determined using one‐way ANOVA followed by Tukey's post hoc test; different letters indicate statistically significant differences between groups (*p* < 0.05). Statistical significance in (e) was determined by Student's *t* test (**p < 0.01, ***p < 0.001). ANOVA, analysis of variance. DW, dry weight. PAR, photosynthetically active radiation.

Fe‐dependent responses in *Arabidopsis* leaves are regulated during reproductive developmental stages (Ngigi et al. 2025), underscoring the importance of leaf age in Fe‐related processes. This prompted us to investigate whether Fe accumulation contributes to the control of leaf senescence. We first quantified Fe content in the third and fourth rosette leaves of wild‐type (Col) plants from 18 to 30 DAE with a 6‐day interval. Total Fe concentration gradually increased with leaf age (Figure 1a). Consistently, transcript levels of Fe‐storage protein genes (*FER1*–*FER4*) increased during age‐dependent leaf senescence (Figure 1b), revealing a positive correlation between Fe accumulation and leaf senescence in *Arabidopsis*.

To directly assess the effect of elevated Fe on leaf senescence, detached Col leaves were treated with optimal (100 µM) and supraoptimal (200 or 300 µM) concentrations of ferric sulphate (Endo et al. 2025) for 2 days. Excess Fe treatment led to pronounced leaf senescence symptoms, including chlorophyll loss and severe necrosis, compared with mock‐treated leaves (Figure 1c). Consistent with these visible phenotypes, ETR, a representative physiological marker of leaf senescence, progressively declined with increasing Fe concentration (Figure 1d), and ETR_max_ was significantly lower in Fe‐treated leaves than in controls (Figure 1e). Transcript levels of senescence marker genes—*ORE1*, *SENESCENCE‐ASSOCIATED GENE 13* (*SAG13*), and *SENESCENCE 4* (*SEN4*)—were significantly increased, whereas those of photosynthetic genes (*CHLOROPHYLL A/B BINDING PROTEIN 1* [*CAB1*] and *CAB4*) were markedly reduced after treatment with 200 μM ferric sulphate for 1 day (Figure S1), demonstrating that excess Fe accelerates leaf senescence in *Arabidopsis*.

To further explore the molecular basis of excess Fe‐induced leaf senescence, we performed mRNA‐seq of Col leaves treated with or without 200 µM ferric sulphate for 1 day. This analysis identified 6150 DEGs in response to Fe treatment (*|*log_2_(FC)| > 0.8, *p* < 0.05) (Supporting Information S1). Comparison with the age‐dependent leaf senescence transcriptome (Woo et al. 2016) revealed substantial overlap; 44.2% of excess Fe‐induced genes (949 of 2148) and 41.3% of excess Fe‐repressed genes (1651 of 4002) were also differentially expressed during age‐dependent senescence (Figure S2). GO enrichment analysis showed that the Fe‐induced DEGs were associated with leaf senescence and stress‐responsive hormone signalling pathways, including responses to ABA, JA, and ethylene (Figure 1f). In contrast, Fe‐repressed DEGs were enriched in chloroplast‐related functions, such as photosynthesis and light responses (Figure 1g). These results suggest that the transcriptomic response to excess Fe shares core molecular programs with age‐dependent leaf senescence. Taken together, these findings establish excess Fe as a potent inducer of premature leaf senescence and support the idea that age‐associated Fe accumulation contributes to triggering the senescence program in *Arabidopsis* leaves.

### 3.2 *FRO6* Is Differentially Expressed in Response to Fe Treatment and During Leaf Senescence

**Figure 2:**
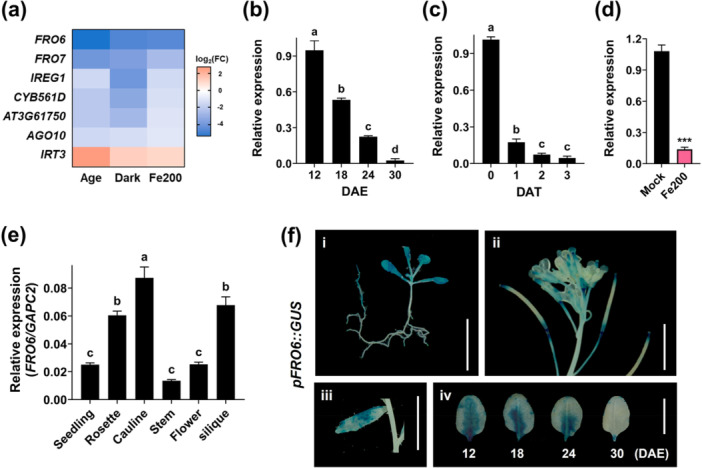
Expression profiles of the *FRO6* gene. (a) Expression patterns of Fe transport and/or homoeostasis‐associated genes that are differentially expressed during leaf senescence. The heatmap displays log_2_(FC) values for DEG related to Fe transport and homoeostasis. Data were derived from RNA‐seq analyses of the third and fourth rosette leaves during age‐dependent (12–30 DAE; Woo et al. 2016), dark‐induced (0–3 days after treatment, DAT; H. Kim, Kim, Vu, et al. 2018), and excess Fe‐induced (mock vs. 200 µM ferric sulphate treatment; Supporting Information S1) senescence. (b–d) Relative transcript levels of *FRO6* during leaf senescence induced by age (b), darkness (c), and excess Fe (d). Transcript levels were quantified by RT‐qPCR in third and fourth leaves of Col plants during age‐dependent (12–30 DAE), dark‐induced (0–3 DAT), and excess Fe‐induced (200 µM ferric sulphate for 1 day) senescence. Expression levels were normalized to *GAPC2* and calculated relative to the respective reference samples. (e) Spatial expression profile of *FRO6*. *FRO6* transcript levels were quantified by RT‐qPCR in various organs, including 2‐week‐old seedlings as well as rosette leaves, cauline leaves, stems, flowers, and siliques from 5 to 6‐week‐old Col plants, and were normalized to *GAPC2*. (f) Histochemical analysis of *FRO6* promoter activity. β‐Glucuronidase (GUS) activity driven by the *FRO6* promoter was examined in (i) 2‐week‐old *pFRO6::GUS* seedlings, (ii) flowers and siliques from 6‐week‐old plants, (iii) cauline leaves and stems from 5‐week‐old plants and (iv) third and fourth rosette leaves at the indicated DAE. Data are presented as mean ± SE of four independent biological replicates for (b–e). Statistical analysis for (b, c, and e) was performed using one‐way ANOVA followed by Tukey's post hoc test; different letters indicate statistically significant differences between groups. Statistical significance for (d) was determined by Student's *t* test (***p < 0.001). ANOVA, analysis of variance; DAE, days after emergence; DEGs, differentially expressed genes; FC, fold‐change; RNA‐seq, RNA‐sequencing; RT‐qPCR, reverse transcription quantitative polymerase chain reaction; Col, Columbia. [Color figure can be viewed at wileyonlinelibrary.com]

Given that Fe concentration gradually increased with leaf age and that excess Fe‐induced physiological and molecular changes resembling leaf senescence (Figures 1 and S2), we hypothesized that Fe transport– and/or Fe homoeostasis–associated proteins contribute to the regulation of senescence. As the first step to test this, we analyzed the transcript levels of 53 corresponding genes across three transcriptome data sets: age‐dependent (12–30 DAE; Woo et al. 2016), dark‐induced (0–3 DAT; H. Kim, Kim, Vu, et al. 2018), and excess Fe‐induced (0 vs. 200 µM ferric sulphate treatment; Supporting Information S1) leaf senescence transcriptomes. Among the 53 genes, one was upregulated and six were downregulated in all three transcriptomes (Figures 2a and Table S2). Of these, *FRO6*, which encodes a plasma membrane‐localized ferric‐chelate reductase (Jain et al. 2014), was prioritized for further analysis due to its marked alteration in expression.

A gradual reduction of *FRO6* transcript levels during both age‐dependent and dark‐induced leaf senescence and decreased expression under excess Fe‐induced leaf senescence was validated by RT‐qPCR (Figure 2b–d). Additional expression profiling revealed that transcript levels of *FRO6* were higher in rosette leaves, cauline leaves, and siliques than in seedlings, stems, and flowers (Figure 2e). To assess the spatial and temporal expression pattern of *FRO6*, we generated transgenic plants harbouring the *GUS* reporter gene driven by the *FRO6* promoter (*pFRO6::GUS*). In seedlings, GUS stain was predominantly detected in shoots rather than roots (Figure 2f(i)). In 5–6‐week‐old plants, strong GUS activity was observed in the cauline leaves, the floral apexes and bases (including pedicels of siliques), as well as sepals and stamens, whereas little or no staining was detected in stems and petals (Figure 2f(ii,iii)). Furthermore, GUS stain in third and fourth rosette leaves gradually decreased from 12 to 30 DAE, with consistently strong signal around the midvein (Figure 2f(iv)). These GUS expression patterns were further validated by RT‐qPCR analysis of localized tissues; *FRO6* transcripts were highly enriched in the shoots of 2‐week‐old seedlings, the medial region of leaves at 12 DAE, and the apical and basal regions of siliques from 6‐week‐old plants (Figure S3). Collectively, these results demonstrate that *FRO6* exhibits dynamic expression patterns under various senescence‐inducing conditions, supporting its potential role in regulating leaf senescence.

### 3.3 FRO6 Functions as a Negative Regulator of Leaf Senescence

**Figure 3:**
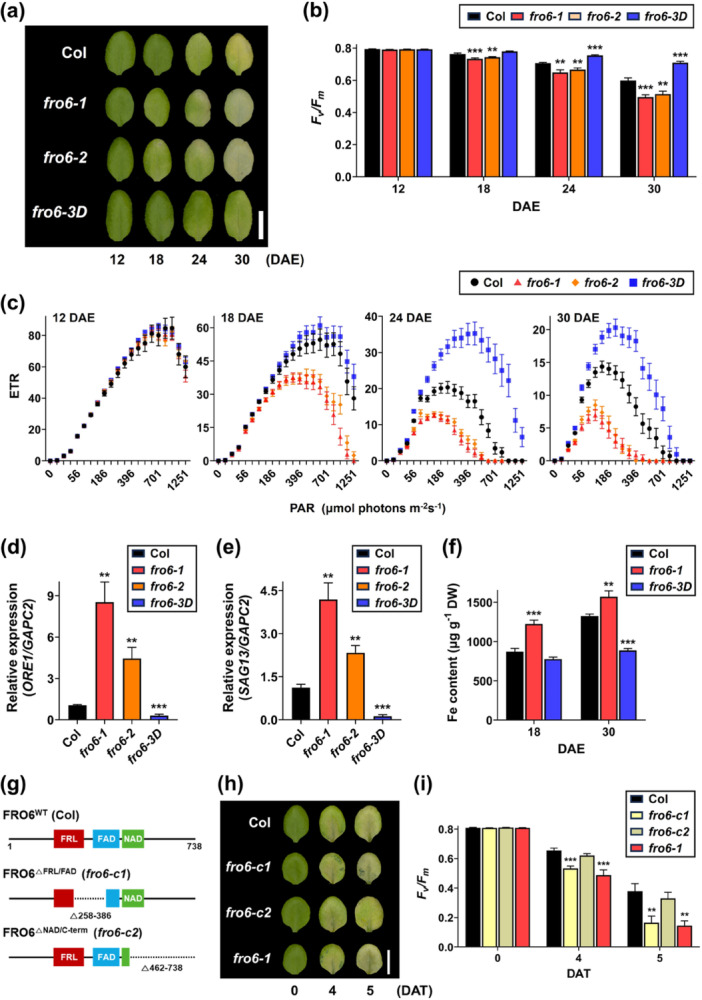
FRO6 as a negative regulator of age‐dependent leaf senescence. (a–c) Age‐dependent leaf senescence phenotypes of the third and fourth rosette leaves from Col, *fro6‐1, fro6‐2* and *fro6‐3D* plants at 12, 18, 24 and 30 DAE. Representative images (a), photochemical efficiency (*F*_v_/*F*_m_) (b), and ETR curves (c) are shown. Scale bar, 1 cm. (d, e) Transcript levels of *ORESARA1* (*ORE1*) (d) and *SENESCENCE‐ASSOCIATED GENE 13* (*SAG13*) (e) determined by RT‐qPCR. RNA was extracted from the third and fourth rosette leaves of Col and *fro6* mutants at 24 DAE. Expression levels were normalized relative to *GAPC2*, and are shown relative to Col. (f) Total Fe content quantification in leaves. Total Fe content in the third and fourth rosette leaves of Col, *fro6‐1* and *fro6‐3D* plants was quantified at 18 and 30 DAE using the bathophenanthroline disulfonate (BPDS) assay. (g) Schematic representation of the major domains of FRO6 and its domain‐specific CRISPR–Cas9 mutants. The diagram illustrates the functional domains in Col and two targeted deletion mutants (*fro6‐c1* and *fro6‐c2*). Dashed lines indicate the deleted regions. FRL, Ferric Reductase transmembrane component‐Like domain; FAD, FAD‐binding domain; NAD, NAD‐binding domain. (h, i) Dark‐induced leaf senescence phenotypes of CRISPR–Cas9‐generated mutants. Representative images (h) and *F*_v_/*F*_m_ (i) of Col, *fro6‐c1*, *fro6‐c2* and *fro6‐1* leaves incubated in continuous darkness for 0, 4 and 5 DAT. Data are presented as mean ± SE (*n* = 20 for b, c; *n* = 8 for f; *n* = 10 for i) and are representative of three independent experiments. Data for (d, e) are presented as mean ± SE of four independent biological replicates. Statistical significance was determined by Student's *t* test (**p < 0.01, ***p < 0.001). Col, Columbia; DAE, days after emergence; DAT, days after treatment; ETR, electron transport rate; FRO6, Ferric Reduction Oxidase 6; RT‐qPCR, reverse transcription quantitative polymerase chain reaction. [Color figure can be viewed at wileyonlinelibrary.com]

To investigate the role of FRO6 in the leaf senescence, we isolated three T‐DNA insertion mutants: *fro6‐1* (loss‐of‐function), *fro6‐2* (partial loss‐of‐function), and *fro6‐3D* (gain‐of‐function with approximately 3.5‐fold higher *FRO6* transcript levels) (Figure S4a; see Section 2 for details). Senescence phenotypes of the third and fourth leaves from Col, *fro6‐1, fro6‐2* and *fro6‐3D* plants were examined at 6‐day intervals from 12 to 30 DAE. As shown in Figure 3a, *fro6‐1* and *fro6‐2* leaves turned yellow earlier, while *fro6‐3D* leaves showed delayed yellowing compared with Col. Consistently, both *F*_v_/*F*_m_ and ETR values in both *fro6‐1* and *fro6‐2* leaves declined more rapidly from 18 DAE onward, whereas *fro6‐3D* retained higher values than Col up to 30 DAE (Figures 3b,c and S4b). In addition, we generated transgenic plants overexpressing *FRO6* under the control of the *CsVMV* promoter (*FRO6‐OE*) to validate the delayed senescence phenotype observed in *fro6‐3D*. Similar to *fro6‐3D*, two independent *FRO6‐OE* lines exhibited delayed age‐dependent leaf senescence (Figure S5).

In accordance with these physiological changes, the expression of senescence marker genes was elevated in *fro6‐1* and *fro6‐2*, but repressed in *fro6‐3D* compared with Col in the third and fourth rosette leaves at 24 DAE (Figure 3d,e). For example, transcript levels of *ORE1* and *SAG13* in *fro6‐1* at 24 DAE were 8.1‐ and 3.7‐fold higher, respectively, than those in Col, whereas their levels in *fro6‐3D* reached only 29.7% and 11.5% of Col levels, respectively. It is noteworthy that *fro6‐1* and *fro6‐3D* leaves were smaller and larger than those of Col, respectively (Figure 3a). In addition, *fro6‐1*and *fro6‐2* flowered earlier, whereas *fro6‐3D* flowered later than Col (Figure S4c). These findings indicate that FRO6 acts as a negative regulator of age‐dependent leaf senescence.

We then assessed dark‐ and excess Fe‐induced leaf senescence using detached third and fourth leaves of *fro6‐1*, *fro6‐2* and *fro6‐3D*. Under both continuous darkness and 200 µM ferric sulphate treatment conditions, *F*_v_/*F*_m_ and ETR values were significantly lower in *fro6‐1* and *fro6‐2* relative to Col, whereas *fro6‐3D* maintained substantially higher values (Figures S6 and S7). Specifically, during dark‐induced senescence, the *F*_v_/*F*_m_ ratios at 4 DAT were 0.197, 0.264 and 0.602 in *fro6‐1, fro6‐2* and *fro6‐3D*, respectively, compared with 0.476 in Col (Figure S6a). A similar trend was observed under excess Fe‐induced senescence, where the *F*_v_/*F*_m_ values at 3 DAT were 0.461, 0.485 and 0.693 in *fro6‐1, fro6‐2* and *fro6‐3D*, respectively, compared with 0.600 in Col (Figure S7a). These results suggest that FRO6 contributes to the regulation of dark‐ and excess Fe‐induced leaf senescence.

To further establish the physiological relevance of FRO6 across a broader range of Fe availability, we extended our analysis to LF conditions using whole plants. Since systemic nutrient redistribution and signalling are critical under Fe deficiency, we conducted these assays in planta on precisely formulated LF agar media or in a soil‐based system (mimicking alkaline Fe deficiency via CaCO_3_ treatment). Under these whole‐plant LF conditions, *fro6‐3D* consistently maintained higher photosynthetic efficiency relative to Col in both agar‐ and soil‐based systems, while *fro6‐1* showed enhanced sensitivity only on LF medium (Figure S8). These findings demonstrate that FRO6 is required for modulating leaf senescence across a broad spectrum of Fe availability—ranging from deficiency to excess.

While these findings demonstrate that FRO6 regulates leaf senescence across various Fe regimes, it remains unclear whether this regulation is directly mediated by its intrinsic ferric reductase activity. To test whether the enzymatic function of FRO6 is indispensable for its role in senescence, we generated domain‐specific CRISPR–Cas9 mutants targeting either the catalytic core (the Ferric Reductase transmembrane component‐Like and FAD‐binding domains, *fro6‐c1*) or the NAD‐binding domain along with the C‐terminal region (*fro6‐c2*) (Figures 3g and S9a–c). In dark‐induced leaf senescence assays, *fro6‐c1* exhibited an early senescence phenotype comparable to that of *fro6‐1*, whereas *fro6‐c2* did not show any significant senescence phenotypes (Figures 3h,i and S9d). These results provide evidence that the catalytic core of FRO6 is indispensable for its function in modulating leaf senescence, supporting the link between its enzymatic potential and the senescence process.

Given the established role of FRO6 in Fe homoeostasis (J. Jeong and Connolly 2009), together with the altered senescence phenotypes observed in the *fro6* mutants under both excess and low Fe conditions (Figures S7 and S8), we examined Fe accumulation in the leaves of the *fro6* mutants. Quantitative BPDS assays revealed that *fro6‐1* leaves accumulated significantly higher Fe contents than the Col at both 18 and 30 DAE, while *fro6‐3D* maintained lower Fe levels (Figure 3f). Together, these findings demonstrate that FRO6 plays an important role in negatively regulating leaf senescence in *Arabidopsis*, likely by modulating Fe accumulation and distribution.

### 3.4 JA Signalling Is Involved in the FRO6‐Mediated Regulation of Leaf Senescence

**Figure 4:**
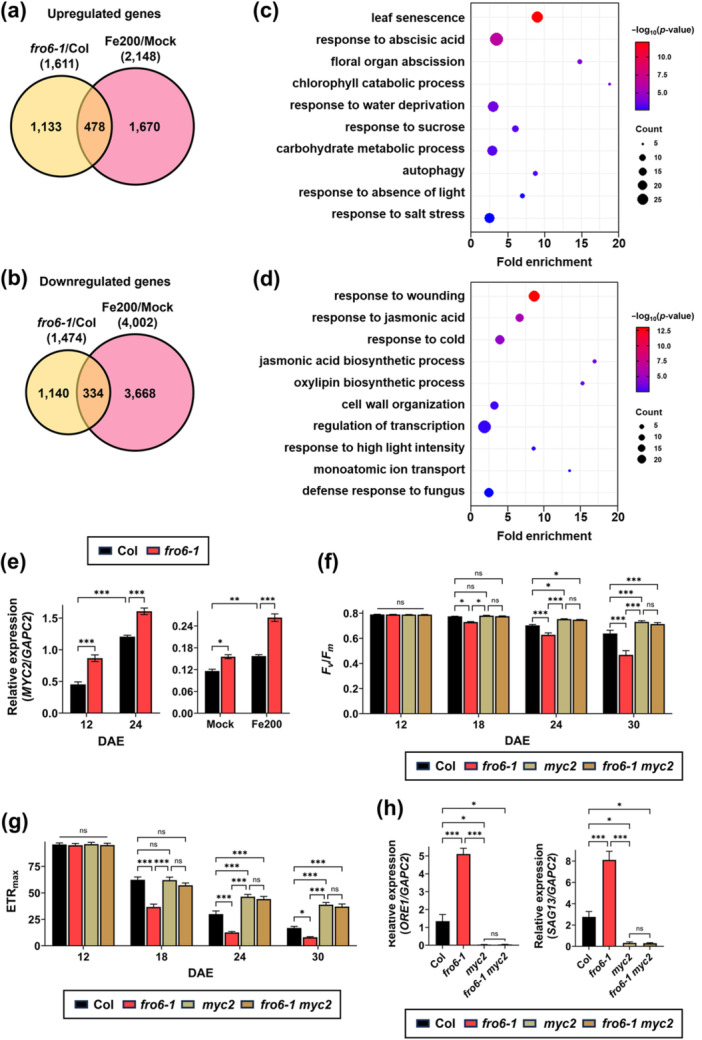
MYC2 is a downstream component of FRO6 in the control of leaf senescence. (a, b) Integrated transcriptomic analysis of FRO6‐regulated and Fe‐responsive gene networks. Venn diagrams show the overlap of upregulated (a) and downregulated (b) genes between FRO6‐regulated DEGs (comparison of Col and *fro6‐1* leaves at 24 DAE, Supporting Information S2) and Fe‐responsive DEGs (Supporting Information S1). DEGs were determined using a threshold of |log_2_(FC)| > 0.8, with *p* < 0.05. (c, d) GO enrichment analysis of overlapping DEG sets. GO enrichment analysis of biological process categories was performed for the genes commonly responsive to both the *fro6‐1* mutation and Fe treatment. The top 10 enriched terms are shown for upregulated (c) and downregulated (d) overlapping DEG sets. Dot size indicates the number of intersection genes, and colour intensity reflects the significance (−log_10_[adjusted *p* value]). (e) Transcriptional regulation of *MYC2* by FRO6 during leaf senescence. *MYC2* transcript levels were quantified by RT‐qPCR in third and fourth leaves of Col and *fro6‐1* plants during age‐dependent (12 and 24 DAE) and excess Fe‐induced (200 µM ferric sulphate for 1 day) senescence. Expression levels were normalized to *GAPC2*. (f–h) Genetic interaction between *FRO6* and *MYC2* in regulating leaf senescence. *F*_v_/*F*_m_ (f) and ETR_max_ (g) were measured in the third and fourth rosette leaves of Col, *fro6‐1*, *myc2* and *fro6‐1 myc2* plants during age‐dependent senescence. (h) Relative expression levels of *ORE1* and *SAG13* were analyzed by RT‐qPCR and normalized to *GAPC2*. Total RNA was extracted from the detached third and fourth leaves at 24 DAE. Data for (f, g) are presented as mean ± SE (*n* = 10) and are representative of three independent trials. Data for (e, h) are presented as mean ± SE of independent biological replicates (*n* = 3 for e; *n* = 4 for h). Statistical analysis for (e–h) was performed using two‐way ANOVA followed by Tukey's multiple comparison test (*p < 0.05, **p < 0.01, ***p < 0.001; ns, not significant). ANOVA, analysis of variance; DAE, days after emergence; DEGs, differentially expressed genes; ETR, electron transport rate; FC, fold‐change; FRO6, Ferric Reduction Oxidase 6; GO, Gene Ontology; RT‐qPCR, reverse transcription quantitative polymerase chain reaction. [Color figure can be viewed at wileyonlinelibrary.com]

To further elucidate the molecular basis underlying FRO6‐mediated control of leaf senescence, we conducted mRNA‐seq using the third and fourth leaves from Col and *fro6‐1* plants at 24 DAE. This analysis identified 1611 upregulated and 1474 downregulated DEGs in *fro6‐1* leaves compared with Col (*|*log_2_(FC)| > 0.8, *p* < 0.05; Supporting Information S2). Comparative analysis with the Fe‐responsive transcriptome (Supporting Information S1) revealed substantial overlap, with 478 commonly upregulated and 334 commonly downregulated DEGs (Figure 4a,b). GO enrichment analysis showed that the commonly upregulated DEGs were enriched in biological processes related to leaf senescence, response to ABA, chlorophyll catabolic process, response to sucrose, and autophagy (Figure 4c). Conversely, the commonly downregulated DEGs were predominantly associated with JA‐related categories, including response to wounding, response to JA, and JA/oxylipin biosynthetic processes (Figure 4d). These findings suggest that *fro6‐1* and Fe‐treated leaves share transcriptional signatures linked to leaf senescence and JA signalling.

On the basis of the enrichment of JA‐related GO terms among the commonly downregulated DEGs (Figure 4d), we next examined the expression profiles of genes involved in JA biosynthesis and the JA‐mediated signalling (Figure S10). Several key JA biosynthetic genes, including *ALLENE OXIDE CYCLASE 1*/*2*/*3* (*AOC1*/*2*/*3*), *ALLENE OXIDE SYNTHASE* (*AOS*) and *12‐OXOPHYTODIENOATE REDUCTASE 3* (*OPR3*), showed reduced transcript levels in both transcriptomes (Figure S10a). Notably, most members (9 of 13) of the *JASMONATE ZIM‐DOMAIN* (*JAZ*) gene family—major repressors of the JA signalling (Han et al. 2023)—were downregulated in the *fro6‐1* transcriptome (Figure S10b).

Since MYC2, a master regulator of JA‐induced senescence, is directly inhibited by JAZ repressors through physical interaction (Chini et al. 2009; Pauwels and Goossens 2011), we hypothesized that FRO6 influences MYC2 activity, potentially at the transcriptional level. To test this hypothesis, we analyzed *MYC2* expression during age‐dependent and Fe‐induced leaf senescence in Col and *fro6‐1*. *MYC2* transcript levels increased significantly under both conditions and were further elevated in *fro6‐1* compared with Col (Figure 4e). These data suggest that the accelerated senescence in *fro6‐1* is associated with the transcriptional up‐regulation of *MYC2*, likely due to the reduced expression of its negative regulators, the *JAZ* genes.

To further investigate the genetic relationship, we generated the *fro6‐1 myc2* double mutant and assessed age‐dependent leaf senescence phenotypes from 12 to 30 DAE. In line with its established role in JA‐mediated senescence (Y. Zhang et al. 2020), the *myc2* mutant delayed age‐dependent leaf senescence (Figure 4f–h). Interestingly, physiological and molecular parameters in *fro6‐1 myc2* were similar to those in *myc2*, but differed significantly from *fro6‐1* at all examined time points. For example, the ETR_max_ values (shown as µmol e^−^ m^−2^ s^−1^) measured at 24 DAE were 29.9 in Col, 12.6 in *fro6‐1*, 46.4 in *myc2* and 44.1 in *fro6‐1 myc2* (Figure 4g). Similarly, transcript levels of *ORE1* and *SAG13* in *fro6‐1 myc2* were comparable to those in *myc2* (Figure 4h). Collectively, these results indicate that *MYC2* acts genetically downstream of *FRO6* in regulating leaf senescence.

### 3.5 SVP Directly Activates *FRO6* Transcription

**Figure 5:**
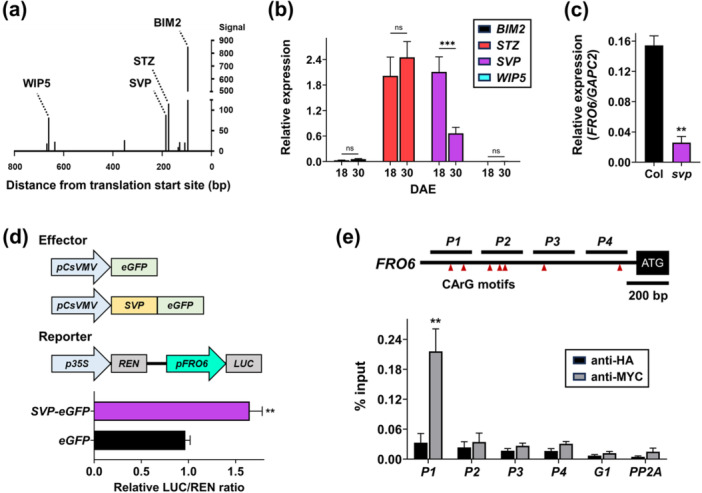
SVP acts as an upstream regulator of *FRO6* expression. (a) Identification of transcription factor (TF) binding sites in the *FRO6* promoter using DNA affinity purification sequencing (DAP‐seq). Schematic representation of DAP‐seq binding sites and enrichment signals within the *FRO6* promoter region. Four TFs—BIM2 (BES1‐INTERACTING MYC‐LIKE2), STZ (SALT TOLERANCE ZINC FINGER), SVP (SHORT VEGETATIVE PHASE) and WIP5 (WIP DOMAIN PROTEIN 5)—exhibited strong binding signals (enrichment signal > 80). (b) Age‐dependent expression patterns of candidate TF genes. Relative transcript levels of *BIM2, STZ, SVP* and *WIP5* were determined by RT‐qPCR in the third and fourth rosette leaves of Col at 18 and 30 DAE. (c) Transcript levels of *FRO6* in the *svp* mutant. *FRO6* expression was determined in the third and fourth rosette leaves of Col and the *svp* mutant at 12 DAE. Expression levels in (b, c) were normalized to *GAPC2*. (d) SVP‐dependent activation of the *FRO6* promoter in dual‐luciferase reporter assays. The relative LUC/REN ratio was quantified in *Arabidopsis* protoplasts. The schematic illustrates the construct used: the reporter contained the *Firefly luciferase* (*LUC*) driven by the *FRO6* promoter and *Renilla luciferase* (*REN*) driven by the *35S* promoter (internal control). The effector contained *SVP* fused with *eGFP* under the *CsVMV* promoter; an empty vector (EV) served as the negative control. Data were normalized to the EV control. (e) Chromatin immunoprecipitation (ChIP)‐qPCR analysis of SVP binding to the *FRO6* promoter. The schematic indicates putative SVP binding motifs in the *FRO6* promoter: *P1* (–1086 to –888), *P2* (–715 to –489), *P3* (–478 to –276) and *P4* (–276 to –30), relative to the translational start codon (ATG). Triangles indicate the positions of CArG motifs. *Protein Phosphatase 2A* (*PP2A*) and *G1* (+1205 to +1354) served as internal controls. ChIP‐qPCR was conducted in *35S::SVP‐MYC* transgenic lines using anti‐MYC antibodies; anti‐HA antibodies were included to assess background binding. Data for (b–e) are presented as mean ± SE of independent biological replicates (*n* = 3 for b; *n* = 4 for c and d; *n* = 5 for e). Statistical significance for (b–e) was determined by Student's *t* test (**p < 0.01, ***p < 0.001; ns, not significant). Col, Columbia; DAE, days after emergence; FRO6, Ferric Reduction Oxidase 6; RT‐qPCR, reverse transcription quantitative polymerase chain reaction. [Color figure can be viewed at wileyonlinelibrary.com]

To identify upstream transcriptional regulators of *FRO6*, we conducted an in silico analysis of published DAP‐seq data to predict TFs that potentially bind to the *FRO6* promoter. Binding peaks of four TFs (BES1‐Interacting Myc‐like2 [BIM2], Salt Tolerance Zinc finger [STZ], SVP, and WIP domain protein 5 [WIP5]) were significantly enriched within the 1‐kb promoter region of *FRO6* (Figure 5a). When their expression profiles were checked at 18‐ and 30‐DAE leaves, only *SVP* showed a decreasing expression pattern that closely resembled that of *FRO6* (Figure 5b). On the basis of this similarity, we focused on SVP, a MADS‐domain TF known to regulate flowering time (Hartmann et al. 2000; Aerts et al. 2018), to investigate its potential role in controlling *FRO6* expression.

To test whether SVP modulates *FRO6* expression, we first measured the transcript level of *FRO6* in the *svp* mutant, which was reduced to 16.8% of Col levels (Figure 5c). We then performed dual‐luciferase assays using an effector construct harbouring the full‐length *SVP* complementary DNA fused to *eGFP* under the control of the *CsVMV* promoter and a reporter construct containing the *FRO6* promoter fused to *LUC* (Figure 5d). Expression of *SVP‐eGFP* enhanced LUC reporter activity (LUC/REN ratio) by approximately 1.7‐fold compared with the empty vector control, indicating that SVP activates the *FRO6* promoter.

Next, we performed ChIP assays using transgenic plants overexpressing SVP fused to a MYC tag, to further validate its association with the *FRO6* promoter. The *FRO6* promoter contains seven canonical CArG motifs (C/AT‐rich/G motifs), typical SVP–binding sites (Z. Wang et al. 2018), distributed across four regions (*P1*–*P4*) (Figure 5e). ChIP‐qPCR analysis revealed significant enrichment of SVP at the *P1* region of the *FRO6* promoter (Figure 5e). Together, these results demonstrate that SVP binds to and activates the *FRO6* promoter.

### 3.6 SVP Regulates Floral Transition and Leaf Senescence by Acting Upstream of *FRO6*

**Figure 6:**
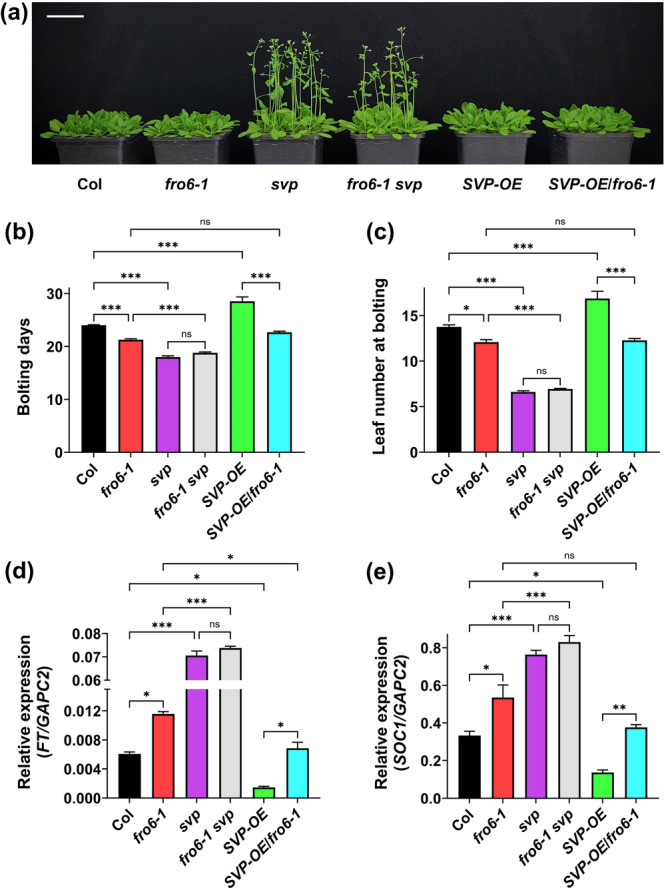
Genetic interactions between *FRO6* and *SVP* in the control of flowering time. (a–c) Flowering‐time phenotypes of Col, *fro6‐1, svp, fro6‐1 svp* double mutant and *SVP* overexpressors in both Col (*SVP‐OE*) and *fro6‐1* mutant (*SVP‐OE*/*fro6‐1*) backgrounds. Representative images of 3‐week‐old whole plants (a), number of days to bolting (b), and total leaf numbers at the time of bolting (c) are shown. Scale bar, 5 cm. Data are presented as mean ± SE (*n* = 15) and are representative of three independent experiments. (d, e) Expression of key flowering integrator genes. Relative expression levels of *FLOWERING LOCUS T* (*FT*) (d) and *SUPPRESSOR OF OVEREXPRESSION OF CONSTANS 1* (*SOC1*) (e) were analyzed by RT‐qPCR and normalized to *GAPC2*. Total RNA was extracted from the detached third and fourth rosette leaves at 12 DAE. Data are presented as mean ± SE of three independent biological replicates. Statistical analysis for (b–e) was determined using two‐way ANOVA followed by Tukey's multiple comparison test (*p < 0.05, **p < 0.01, ***p < 0.001; ns, not significant). ANOVA, analysis of variance; Col, Columbia; DAE, days after emergence; FRO6, Ferric Reduction Oxidase 6; RT‐qPCR, reverse transcription quantitative polymerase chain reaction; SVP, Short Vegetative Phase. [Color figure can be viewed at wileyonlinelibrary.com]

As SVP is a key repressor of floral induction (Y. Wang, Severing, et al. 2020), we first investigated whether the SVP–FRO6 regulatory module contributes to floral transition. Flowering phenotypes and the expression of floral integrator genes were analyzed in *fro6‐1*, *svp* and *fro6‐1 svp* mutants, as well as in *SVP* overexpressing transgenic plants (*SVP‐OE* and *SVP‐OE*/*fro6‐1*), under LD conditions. Consistent with previous reports (Fujiwara et al. 2008; H. G. Lee, Kim, et al. 2025), *svp* and *SVP‐OE* plants exhibited early and delayed flowering, respectively (Figures 6a–c and S11). In addition, *fro6‐1* plants flowered slightly earlier than Col. We next measured transcript levels of two floral integrator genes, *FT* and *SOC1*, both of which are direct targets of SVP (J. H. Lee et al. 2007; D. Li et al. 2008). In agreement with their flowering phenotypes, *svp* and *fro6‐1* exhibited elevated expressions of *FT* and *SOC1*, whereas *SVP‐OE* plants displayed reduced transcript levels (Figure 6d,e).

Notably, both the flowering time (days to bolting and rosette leaf number at bolting) and the expression of *FT* and *SOC1* in the *fro6‐1 svp* double mutant were comparable to those of *svp* but clearly distinct from *fro6‐1* (Figure 6b–e). In contrast, the delayed flowering phenotype of *SVP‐OE* was abolished in *SVP‐OE*/*fro6‐1*, which flowered similarly to *fro6‐1*. The average rosette leaf numbers at bolting were 16.9 ± 0.8 for *SVP‐OE*, 12.3 ± 0.2 for *SVP‐OE*/*fro6‐1* and 12.1 ± 0.3 for *fro6‐1*. Likewise, the suppression of *FT* and *SOC1* observed in *SVP‐OE* was alleviated in *SVP‐OE*/*fro6‐1* (Figure 6d,e), indicating that *SVP* overexpression failed to further repress *FT* and *SOC1* in the absence of functional FRO6. These results suggest that the SVP–FRO6 regulatory module contributes to the control of floral transition by modulating *FT* and *SOC*, with FRO6 acting downstream of SVP.

**Figure 7:**
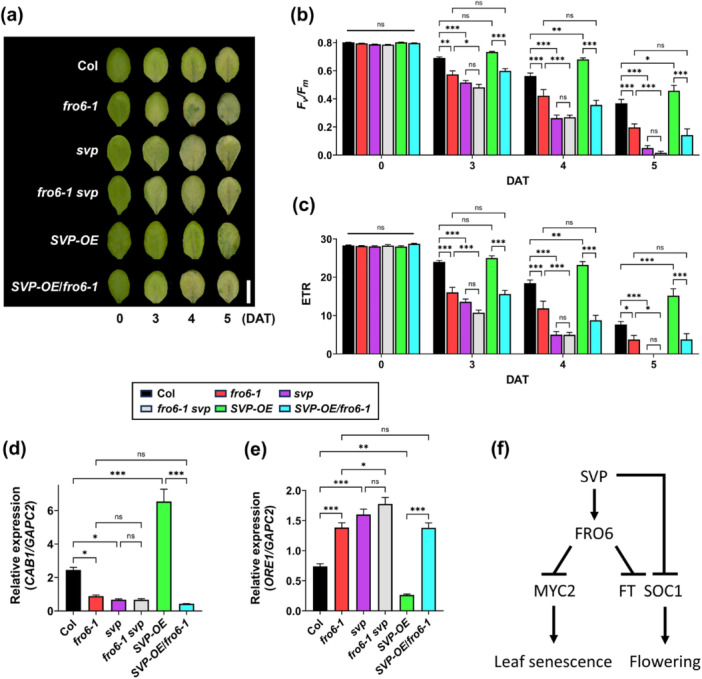
Genetic interaction defining the SVP–FRO6 regulatory module in leaf senescence. (a–c) Dark‐induced leaf senescence phenotypes of third and fourth rosette leaves from Col, *fro6‐1, svp, fro6‐1 svp*, *SVP‐OE* and *SVP‐OE*/*fro6‐1* at 0, 3, 4 and 5 DAT. Representative images (a), photochemical efficiency (*F*_v_/*F*_m_) (b), and ETR (c) are shown. Scale bar, 1 cm. Data are presented as mean ± SE (*n* = 15) and are representative of three independent experiments. (d, e) Expression analysis of senescence‐marker genes. Relative transcript levels of *CHLOROPHYLL A/B‐BINDING PROTEIN 1* (*CAB1*) (d) and *ORE1* (e) were analyzed by RT‐qPCR and normalized to *GAPC2*. Total RNA was extracted from the detached third and fourth leaves (collected at 12 DAE) following 2 days of dark incubation. Data are presented as mean ± SE of three independent biological replicates. Statistical analysis for (b–e) was performed using two‐way ANOVA followed by Tukey's multiple comparison test (*p < 0.05, **p < 0.01, ***p < 0.001; ns, not significant). (f) Proposed model of the SVP–FRO6 regulatory module. Schematic representation of the SVP–FRO6 regulatory hierarchy: SVP directly activates *FRO6* expression. FRO6, in turn, acts as a negative regulator of both leaf senescence and flowering by repressing the jasmonic acid (JA) signalling–associated TF MYC2, as well as the key flowering integrators FT and SOC1. Thus, the SVP–FRO6 regulatory module ensures the coordinated timing of leaf senescence and reproductive development in *Arabidopsis*. ANOVA, analysis of variance; Col, Columbia; DAE, days after emergence; DAT, days after treatment; ETR, electron transport rate; FRO6, Ferric Reduction Oxidase 6; RT‐qPCR, reverse transcription quantitative polymerase chain reaction; SVP, Short Vegetative Phase; TFs, transcription factors. [Color figure can be viewed at wileyonlinelibrary.com]

Lastly, we examined the genetic relationship between *SVP* and *FRO6* in the regulation of dark‐induced leaf senescence. Compared with Col, *svp* leaves showed accelerated declines in *F*_v_/*F*_m_ and ETR, whereas *SVP‐OE* leaves exhibited delayed declines (Figure 7a–c). Consistent with these physiological changes, expression of *CAB1* was significantly decreased in *svp* but remained at higher levels in *SVP‐OE* at 2 DAT, while *ORE1* expression showed the opposite trend (Figure 7d,e). The *fro6‐1 svp* double mutant displayed accelerated senescence phenotypes comparable to *svp*, which were more pronounced than those of *fro6‐1* alone (Figure 7a–e). Conversely, the delayed senescence phenotypes observed in *SVP‐OE* were markedly suppressed in *SVP‐OE*/*fro6‐1*, resembling *fro6‐1*. Our data indicate that FRO6 is required for SVP‐mediated suppression of leaf senescence. Overall, these findings reveal that the SVP–FRO6 regulatory module defines a shared regulatory axis contributing to the control of leaf senescence and floral transition (Figure 7f).

## 4 Discussion

Fe is essential for normal plant growth, supporting key enzymatic processes involved in chlorophyll synthesis, photosynthesis, respiration, and nutrient metabolism (Ning et al. 2023). Despite its well‐established biochemical functions, the role of Fe availability in shaping developmental timing—particularly leaf senescence and flowering—remains poorly understood. Here, we demonstrate that endogenous Fe levels progressively increase during age‐dependent leaf senescence and excess Fe treatment accelerates leaf senescence (Figures 1a–e and S1). Transcriptome profiling further reveals a substantial overlap between Fe‐induced and age‐dependent leaf senescence programs (Figures 1f,g and S2). Our study identifies FRO6 as a novel negative regulator of leaf senescence acting through the JA signalling pathway via MYC2, while concurrently delaying floral transition by suppressing *FT* and *SOC1* expression (Figures 3, 4, and 6). Moreover, we show that SVP acts upstream of FRO6 by directly activating its transcription (Figure 5). Taken together, these findings establish the SVP–FRO6 regulatory module as a shared regulatory axis that coordinately restrains both leaf senescence and floral transition (Figure 7f).

### 4.1 Fe Accumulation: A Conserved Metabolic Hallmark of Senescence

Our data strongly support the hypothesis that Fe accumulation promotes leaf senescence in *Arabidopsis*. We initially observed a pronounced age‐dependent increase in total Fe content and in the induction of the iron‐storage protein genes (*FER1‐4*) in senescing leaves (Figure 1a,b). This is consistent with observations in *B. napus*, where supraoptimal Fe treatment causes a pronounced Fe accumulation in leaves (Sági‐Kazár et al. 2021). Crucially, exogenous Fe application accelerated both physiological and molecular changes associated with leaf senescence (Figures 1c–g and S1). Notably, similar phenomena have been reported across mammalian systems. Intracellular Fe accumulation accompanies senescence induction in mouse embryonic fibroblasts and human primary cells (Masaldan et al. 2018), and aged mice exhibit elevated non‐heme Fe levels in tissues like skeletal muscle, which correlate with enhanced ROS production, mitochondrial dysfunction, and age‐related muscle degeneration (Sato et al. 2022; Alves et al. 2023). Overall, these findings suggest that Fe accumulation represents a conserved metabolic hallmark of the aging process across diverse kingdoms.

In this context, the function of FER1, which strongly accumulates under excess Fe conditions (Reyt et al. 2015), is particularly relevant. Loss‐of‐function *fer1* mutants display accelerated senescence phenotypes, indicating that FER1 negatively regulates leaf senescence in *Arabidopsis* (Murgia et al. 2007). Similarly, the H2B histone variant HTB4 acts as a negative regulator of leaf senescence, with a premature senescence phenotype of the *htb4* mutant linked to downregulation of bHLH TFs involved in Fe homoeostasis, leading to reduced expression of Fe uptake machinery components, like *FRO2* and *IRT1* (Yang et al. 2023). Interestingly, exogenous Fe application suppresses the early senescence phenotype of *htb4*, suggesting that reduced Fe availability contributes to its premature senescence. Future studies examining whether overexpression of *FRO6* can rescue premature senescence and altered Fe‐deficiency responses observed in the *fer1* and *htb4* mutants would help clarify the interplay between Fe accumulation and the FRO6‐mediated senescence pathway.

### 4.2 Role of FRO6 and Fe Homoeostasis in Controlling Leaf Senescence

Our results demonstrated that excess Fe accelerates leaf senescence and that Fe content is significantly elevated in the *fro6‐1* mutant (Figures 1a and 3f), strongly implicating FRO6‐mediated Fe regulation in the control of leaf senescence. Given the predominant expression of *FRO6* in aerial tissues (Figure 2e,f; Feng et al. 2006), the elevated Fe levels observed in *fro6‐1* may reflect alterations relevant to Fe uptake, transport, and/or recycling within senescing leaves. While plants possess sophisticated regulatory systems to cope with Fe deficiency (Kobayashi et al. 2019; M. Li et al. 2023), the precise mechanisms governing Fe accumulation and redistribution during leaf senescence—strategic processes for nutrient remobilization—are still not fully understood. Given that Fe accumulation enhances oxidative stress, a hallmark of aging (Lapaz et al. 2022), elucidating the downstream consequences of FRO6‐dependent Fe regulation is critical. To comprehensively understand how Fe status—regulated by FRO6—dictates leaf senescence, future studies should focus on Fe‐dependent metabolic reprogramming. In particular, liquid chromatography–tandem mass spectrometry (LC–MS)/MS‐based metabolomic profiling of *fro6* mutants under sufficient and excess Fe conditions may reveal key changes in antioxidant metabolites, ROS‐related intermediates, and lipid peroxidation products associated with leaf senescence. Such analyses would provide mechanistic insight into how FRO6‐dependent Fe accumulation contributes to oxidative stress during leaf senescence. Furthermore, while our study primarily highlights the detrimental effects of excess Fe, our additional results demonstrate that FRO6 is also essential for maintaining photosynthetic efficiency under LF conditions (Figure S8). This dual role suggests that FRO6 acts as a physiological buffer, optimizing leaf longevity across a wide range of Fe availability.

### 4.3 Distinct Role of FRO6 in Regulating Leaf Senescence

Although the *Arabidopsis* genome encodes eight *FRO* family members, the extent of their functional diversification remains incompletely understood. *FRO6* and *FRO7* are tandemly arranged on chromosome 5 and share high amino acid sequence similarity (Muhammad et al. 2018; Anjali et al. 2021). FRO7 has been reported to play a critical role in seedling survival under Fe‐limiting conditions and is predominantly expressed in young, developing leaves during early seedling stages (J. Jeong et al. 2008). In contrast, our findings support a distinct and nonredundant function for FRO6 as a key regulator of senescence in leaves, consistent with its strong expression in both rosette and cauline leaves (Figure 2e). Future genetic analyses of the *fro6 fro7* double mutant would be informative in determining whether functional redundancy emerges under particular stress conditions or developmental contexts.

### 4.4 FRO6–MYC2‐Mediated Modulation of JA Signalling in Leaf Senescence and Flowering

Transcriptome analysis revealed a strong enrichment of JA‐related GO terms among genes commonly repressed in both the excess Fe–induced and *fro6‐1* transcriptomes (Figure 4d). Several *JAZ* repressors—including *JAZ7*—were markedly downregulated in the *fro6‐1* mutant (Figure S10c). JAZ7 is a key transcriptional repressor of JA signalling that negatively regulates dark‐induced leaf senescence. The loss of JAZ7 function releases MYC2, thereby activating JA‐mediated signalling pathways (Yu et al. 2016). In support of this, we found that *MYC2* transcript levels were significantly elevated in *fro6‐1* compared with Col under both age‐dependent and Fe‐induced senescence conditions (Figure 4e), providing a transcriptional basis for increased MYC2 activity in the absence of FRO6. During JA‐induced leaf senescence, MYC2 promotes H_2_O_2_ accumulation and activates *UP‐REGULATED DURING SENESCENCE 1* (*AtUSR1*), a positive regulator of leaf senescence (Y. Zhang et al. 2020). Consistent with this regulatory framework, the *myc2* mutant delayed age‐dependent leaf senescence phenotype, and genetic analyses placed MYC2 downstream of FRO6 (Figure 4e–h). MYC2 also mediates JA‐dependent suppression of *FE‐DEFICIENCY INDUCED TRANSCRIPTION FACTOR* (*FIT*; *bHLH29*), the master regulator of Fe‐deficiency responses (Y. Cui et al. 2018). Given its dual roles in JA‐mediated leaf senescence and Fe‐deficiency signalling, our data position the FRO6‐MYC2 axis as a key mechanistic link integrating Fe status with JA signalling‐mediated senescence programs.

In addition to its role in leaf senescence, MYC2 influences flowering time. MYC2 directly suppresses *FLC* expression by binding to its promoter, thereby relieving FLC‐mediated repression of FT and promoting flowering (Chakraborty et al. 2025). Consistently, MYC2 overexpression results in an early‐flowering phenotype compared with the wild type (Chakraborty et al. 2025). The transcriptional up‐regulation of MYC2 in *fro6‐1* thus raise the possibility that FRO6 influences the floral transition not only via the FT/SOC1 axis (as demonstrated in this study) but also through MYC2‐dependent JA signalling. Future studies using the *fro6* single and *fro6 myc2* double mutants will clarify whether FRO6 modulates *FLC* expression through the MYC2‐dependent pathway.

### 4.5 SVP–FRO6 Regulatory Module in the Control of Flowering

We demonstrated that SVP acted as a direct upstream activator of *FRO6* expression (Figure 5d,e). Genetic analyses further revealed that FRO6 is required for SVP‐mediated repression of the floral transition under LD conditions (Figure 6b,c). In the photoperiodic flowering pathway, multilayered regulatory interactions between CO and FT are well established, with CO activating SOC1 through FT to promote flowering (Yoo et al. 2005; Takagi et al. 2023). The regulatory relationships linking FRO6 with CO, FT, and SOC1 thus provide new insight into how Fe‐related pathways intersect with canonical flowering networks. Given that Fe deficiency delays flowering (W. Chen et al. 2021), the elevated endogenous Fe content in *fro6‐1* might contribute to its early‐flowering phenotype. Future studies investigating how SVP integrates Fe status into the regulation of flowering will further elucidate the mechanistic connection between Fe homoeostasis and floral transition.

Finally, since other MADS‐box TFs such as AGAMOUS‐LIKE 24 (AGL24), FLC, and MADS AFFECTING FLOWERING 1 (MAF1) bind to CArG motifs of the *FT* and/or *SOC1* promoter (Liu et al. 2008; Gu et al. 2013; J. H. Lee et al. 2013; Posé et al. 2013; Y. Wang, Severing, et al. 2020), it will be valuable to determine whether these MADS‐box TFs also contribute to FRO6‐dependent flowering responses. A deeper understanding of how MADS‐box TFs integrate flowering signals with Fe status will clarify the positioning of FRO6 within the broader flowering regulatory network and its role in optimizing plant fitness.

## Conflicts of Interest

The author declares no conflicts of interest.

## Supporting information


**Figure S1:** Expression analysis of senescence marker and photosynthesis‐related genes upon excess Fe treatment.
**Figure S2:** Comparative transcriptomic analysis of excess Fe‐induced and age‐dependent leaf senescence.
**Figure S3:** Spatial expression analysis of FRO6 in dissected tissues.
**Figure S4:** Phenotypes of *fro6* mutants in leaf senescence and floral transition.
**Figure S5:** Age‐dependent senescence phenotypes of FRO6‐OE lines.
**Figure S6:** Dark‐induced leaf senescence phenotypes of the *fro6* mutants.
**Figure S7:** Excess Fe‐induced leaf senescence phenotypes of the *fro6* mutants.
**Figure S8:** Low‐Fe‐mediated senescence phenotypes of the *fro6* mutants.
**Figure S9:** Altered leaf senescence phenotypes of CRISPR–Cas9‐generated FRO6 deletion mutants.
**Figure S10:** Changes in JA‐associated gene expression in the *fro6‐1* mutant and in excess Fe‐treated leaves.
**Figure S11:** Flowering‐time phenotypes of genetic combinations involving *fro6*, *svp*, and an SVP overexpression line.
**Table S1:** Primers used in this study.
**Table S2:** Expression patterns of Fe transport‐ and/or homeostasis‐associated genes that were differentially expressed during age‐dependent, dark‐induced, and excess Fe‐induced senescence.
**Table S3:** A list of putative binding targets at the FRO6 promoter with their DAP‐seq peak enrichment signals.

Supporting File:

## Data Availability

The mRNA‐seq data generated in this study have been deposited in the European Nucleotide Archive (ENA) under study number PRJEB101033. The data underlying this article are available in the article and in its online supporting material.
