# Supplementary material for: The SVP–FRO6 Module Integrates the Control of Leaf Senescence and Flowering in Arabidopsis
Source: Plant Cell Environ. 2026 Jun 18;49(9):7191–209. doi: 10.1111/pce.70677 (PMC13436468; doi:10.1111/pce.70677)
Supplement: Supplementary file 1 [file PCE-49-7191-s001.docx]

***Plant, Cell & Environment* Supporting Information**

**The SVP–FRO6 module integrates the control of leaf senescence and flowering in *Arabidopsis***

Hyunsu Park^1^, Yeong Seon Yoon^1^, Sanghoon Park^1^, Ukcheol Jeong^1^, Hyeyoung Choi^1^, Hyunwoo Oh^1^, Dovran Shamamedov^1^, Zhonghai Li^2^, and Hye Ryun Woo^1,3,*^

^1^Department of New Biology, Daegu Gyeongbuk Institute of Science and Technology (DGIST), Daegu, 42988, Korea

^2^State Key Laboratory of Tree Genetics and Breeding, College of Biological Sciences and Technology, Beijing Forestry University, Beijing, 100083, China

^3^New Biology Research Center, Daegu Gyeongbuk Institute of Science and Technology (DGIST), Daegu, 42988, Korea

^*^Correspondence: Hye Ryun Woo (hrwoo@dgist.ac.kr)

The following Supporting Information is available for this article:

**Figure S1** Expression analysis of senescence marker and photosynthesis-related genes upon excess Fe treatment.

**Figure S2** Comparative transcriptomic analysis of excess Fe-induced and age-dependent leaf senescence.

**Figure S3** Spatial expression analysis of *FRO6* in dissected tissues.

**Figure S4** Phenotypes of *fro6* mutants in leaf senescence and floral transition.

**Figure S5** Age-dependent senescence phenotypes of *FRO6-OE* lines.

**Figure S6** Dark-induced leaf senescence phenotypes of the *fro6* mutants.

**Figure S7** Excess Fe-induced leaf senescence phenotypes of the *fro6* mutants.

**Figure S8** Low-Fe mediated senescence phenotypes of the *fro6* mutants.

**Figure S9** Altered leaf senescence phenotypes of CRISPR–Cas9-generated *FRO6* deletion mutants.

**Figure S10** Changes in JA-associated gene expression in the *fro6-1* mutant and in excess Fe-treated leaves.

**Figure S11** Flowering-time phenotypes of genetic combinations involving *fro6*, *svp*, and an *SVP* overexpression line.

**Table S1** Primers used in this study.

**Table S2** Expression patterns of Fe transport and/or homeostasis-associated genes that were differentially expressed during age-dependent, dark-induced, and excess Fe-induced senescence.

**Table S3** A list of putative binding targets at the *FRO6* promoter with their DAP-seq peak enrichment signals.

**Figure S1** Expression analysis of senescence marker and photosynthesis-related genes upon excess Fe treatment. (a, b) Transcript levels of senescence marker genes (*ORE1*, *SAG13*, and *SEN4*) (a) and photosynthesis-related genes (*CAB1* and *CAB4*) (b) in the third and fourth rosette leaves of Col treated with or without 200 µM ferric sulfate treatment for 1 day, as measured by RT-qPCR. Expression levels were normalized to *GAPC2* and are presented relative to the mock-treated controls. Mock, no ferric sulfate; Fe200, 200 µM ferric sulfate. Data are presented as mean ± SE of four independent biological replicates. Statistical significance was determined by Student’s t-test (***p* < 0.01; ****p* < 0.001).

**Figure S2** Comparative transcriptomic analysis of excess Fe-induced and age-dependent leaf senescence. (a, b) Venn diagrams illustrating the overlap of upregulated (a) and downregulated (b) DEGs. DEGs responsive to excess Fe treatment (Dataset S1) were compared with those regulated during age-dependent leaf senescence (Woo *et al*., 2016). The number of DEGs was determined using a threshold of |log_2_(FC)| > 0.8 for the Fe-responsive dataset and |log_2_(FC)| > 1.5 for the age-dependent dataset, both with *p* < 0.05.

**Figure S3** Spatial expression analysis of *FRO6* in dissected tissues. (a–c) Transcript levels of *FRO6* were analyzed by RT-qPCR in various tissues and subregions of Col, including: (a) shoot and root from 2-week-old seedlings; (b) medial and lateral regions of the third and fourth rosette leaves at 12 DAE; and (c) basal, medial, and apical regions of siliques from 6-week-old plants. A schematic illustration of the tissue sectioning is provided below the graphs; rosette leaves and siliques were subdivided into representative regions for spatial expression analysis. Expression levels in (a) and (b) were normalized to *GAPC2*, while those in (c) were normalized to *PROTEIN PHOSPHATASE 2A* (*PP2A*). Data are presented as mean ± SE of three independent biological replicates. Statistical significance was determined by Student’s t-test (***p* < 0.01; ****p* < 0.001).

**Figure S4** Phenotypes of *fro6* mutants in leaf senescence and floral transition. (a) Transcript levels of *FRO6* in T-DNA insertion mutants (*fro6-1*, *fro6-2*, and *fro6-3D*) at 24 DAE, quantified by RT-qPCR and normalized to *GAPC2*. Data are presented as mean ± SE of four independent biological replicates. (b) ETR_max_ measured in the third and fourth rosette leaves of Col, *fro6-1*, *fro6-2*, and *fro6-3D* plants during age-dependent senescence (12, 18, 24, and 30 DAE). Data are shown as mean ± SE (*n*=20) and are representative of three independent experiments. (c) Representative flowering phenotypes of 32-day-old Col, *fro6-1*, *fro6-2*, and *fro6-3D* plants. Scale bar, 5 cm. Statistical significance for (a, b) was assessed using Student’s t-test (**p* < 0.05; ***p* < 0.01; ****p* < 0.001).

**Figure S5** Age-dependent senescence phenotypes of *FRO6-OE* lines. (a, b) *F_v_*/*F_m_* (a) and ETR_max_ (b) of Col and *FRO6-OE* lines at 12, 18, 24, and 30 DAE. Data are expressed as mean ± SE (*n*=10) and are representative of three independent experiments. Statistical significance was determined by Student’s t-test (***p* < 0.01; ****p* < 0.001).

**Figure S6** Dark-induced leaf senescence phenotypes of the *fro6* mutants. (a, b) *F_v_*/*F_m_* (a) and ETR (b) of Col, *fro6-1*, *fro6-2*, and *fro6-3D* leaves incubated in continuous darkness for 0, 3, 4, and 5 DAT. Data are expressed as mean ± SE (*n*=10) and are representative of three independent experiments. Statistical significance was determined by Student’s t-test (**p* < 0.05; ***p* < 0.01; ****p* < 0.001).

**Figure S7** Excess Fe-induced leaf senescence phenotypes of the *fro6* mutants. (a, b) *F_v_*/*F_m_* (a) and ETR_max_ (b) of Col, *fro6-1*, *fro6-2*, and *fro6-3D* following incubation in the presence or absence of 200 µM ferric sulfate for 0, 1, 2, and 3 DAT. Data are presented as mean ± SE (*n*=12) and are representative of three independent experiments. Statistical significance was determined by Student’s t-test (**p* < 0.05; ***p* < 0.01; ****p* < 0.001).

**Figure S8** Low-Fe mediated senescence phenotypes of the *fro6* mutants. (a-c) Effect of low-Fe (LF) conditions on *fro6* mutants grown on sufficient-Fe (SF) or LF agar medium. (a) Representative whole plant phenotypes of Col, *fro6-1*, and *fro6-3D* grown for 18 days on SF or LF medium. Scale bar, 1cm. *F_v_*/*F_m_* (b) and ETR (c) measured in the third and fourth rosette leaves of Col, *fro6-1*, and *fro6-3D* plants. (d–f) LF-induced senescence phenotypes of the third and fourth rosette leaves from Col, *fro6-1*, and *fro6-3D* grown for 4 weeks in SF- or LF-soil (see Methods for CaCO₃ treatment). Representative images (d), *F_v_*/*F_m_* (e), and ETR (f) are shown. Scale bar, 1 cm. Data are presented as mean ± SE (n=5 for b, c; n=10 for e, f) and are representative of three independent experiments. Statistical analysis was performed using one-way ANOVA followed by Tukey’s multiple comparison test (**p* < 0.05; ***p* < 0.01; ****p* < 0.001).

**Figure S9** Altered leaf senescence phenotypes of CRISPR–Cas9-generated *FRO6* deletion mutants. (a) Schematic representation of the *FRO6* genomic structure, indicating the positions of guide RNAs (gRNAs) and the deleted regions in *fro6-c1* and *fro6-c2* mutants. Exons and introns are indicated by black and white boxes, respectively. (b) Genomic sequences of *fro6-c1* and *fro6-c2* showing large fragment deletions between the two gRNA target sites (highlighted in yellow and peach). Deletions were validated by Sanger sequencing. (c) Comparison of FRO6 amino acid sequences and functional domains in Col, *fro6-c1*, and *fro6-c2*. Functional domains were identified using InterPro (https://ebi.ac.uk/interpro/). Underlined regions represent the following features: red, Ferric Reductase transmembrane component-Like (FRL) domain; sky blue, FAD-binding domain; green, NAD-binding domain. (d) ETR values of CRISPR-Cas9-generated mutants during dark-induced leaf senescence. ETR values of detached leaves from Col, *fro6-c1*, *fro6-c2*, and *fro6-1* incubated under continuous darkness for 0, 4, and 5 DAT. Data are expressed as mean ± SE (n=10) and are representative of three independent experiments. Statistical significance was determined by Student’s t-test (****p* < 0.001).

**Figure S10** Changes in JA-associated gene expression in the *fro6-1* mutant and in excess Fe-treated leaves. (a, b) Heatmaps illustrating differential expression of genes involved in the JA biosynthetic process and JA-mediated signaling pathway in *fro6-1* relative to Col, as well as under Fe-treated versus mock-treated conditions. The log₂(FC) values for *fro6-1*/Col were derived from RNA-seq analysis of Col and *fro6-1* leaves at 24 DAE, and the log₂(FC) values for Fe200/Mock were obtained from RNA-seq analysis of leaves treated with or without 200 µM ferric sulfate for 1 day. Genes highlighted in red indicate JA-related genes that exhibited reduced expression in both conditions, with log₂(FC) < −0.8 and *p* < 0.05 in the *fro6-1*−mediated transcriptome.

**Figure S11** Flowering-time phenotypes of genetic combinations involving *fro6*, *svp*, and an *SVP* overexpression line. Representative phenotypes of 30-day-old Col, *fro6-1*, *svp*, *fro6-1 svp*, *SVP-OE*, and *SVP-OE*/*fro6-1* plants are shown. Scale bar, 5 cm.

**Table S1** Primers used in this study.

| **Names** | **Sequence (5'-3')** | **Purpose** |
| --- | --- | --- |
| FRO6_dtopo_F | CACCATGGATGATTATGAAACCCCTC | Plasmid construction |
| FRO6_dtopo_R | GAGATCGAAACTGTGGCT | Plasmid construction |
| pFRO6_dtopo_F | CACCCGATGCTCTCAAGGCCAAAGA | Plasmid construction |
| pFRO6_dtopo_R | CTTTATTTGAATTTCCACTTCTCAGTG | Plasmid construction |
| SVP_dtopo_F | CACCATGGCGAGAGAAAAGATTCAGATC | Plasmid construction |
| SVP_dtopo_R | ACCACCATACGGTAAGCCGAG | Plasmid construction |
| FRO6_gRNA1_F | TTTTGGTCTCAATTGATCGGAATTGCTATCTTACCGTTTTAGAGCTAGAAATAGC | Plasmid construction |
| FRO6_gRNA2_R | TTTTGGTCTCAAAACTCTAGATGGGAACCATCATGCAATCACTACTTCGACTC | Plasmid construction |
| FRO6_gRNA3_F | TTTTGGTCTCAATTGGAAAGATTTATTGCGGGTGCGTTTTAGAGCTAGAAATAGC | Plasmid construction |
| FRO6_gRNA4_R | TTTTGGTCTCAAAACTCTGTCAAGCTACCACGGACCAATCACTACTTCGACTC | Plasmid construction |
| Salk_099597_LP | TGTCCAATGTAGAAACACCAACA | genotyping |
| Salk_099597_RP | CATTTAGTGTTTCTTCAAGCCCTC | genotyping |
| CS802040_LP | TTTGTTATTGGATTTTGTTTCGG | genotyping |
| CS802040_RP | ATCATCACCGAGTTGTTCAGG | genotyping |
| Salk_138745_LP | GAAACCCCTCTTTTGTCCAAG | genotyping |
| Salk_138745_RP | CACTGTAACCAAGACAGTTCCTT | genotyping |
| Salk_066466_LP | TCACTCTGCAAACACATCTCG | genotyping |
| Salk_066466_RP | ACGATTTTGGATTTTTGACCC | genotyping |
| Salk_017005_LP | GGCGGGATTTAATCAAGAGAC | genotyping |
| Salk_017005_RP | TTTGGTACAACCGCTCGTAAC | genotyping |
| FER1_qRT_F | CAACGTTGCTATGAAGGGACTAGC | RT-qPCR |
| FER1_qRT_R | ACTCTTCCTCCTCTTTGGTTCTGG | RT-qPCR |
| FER2_qRT_F | TGCCATCAACGATCAGATCAACG | RT-qPCR |
| FER2_qRT_R | AGGCATACAGGGCATGGTAGAC | RT-qPCR |
| FER3_qRT_F | AGTGTGTTTCTGAACGAACAGGTG | RT-qPCR |
| FER3_qRT_R | AGAAGCTCCTGATCGAAATGCC | RT-qPCR |
| FER4_qRT_F | CGAGTTTCTGACAGAGCAGGTG | RT-qPCR |
| FER4_qRT_R | CGTCGCAGTTGAGCCACATATTC | RT-qPCR |
| FRO6_qRT_F | TGTACCCTGAACAACTCGGTGATG | RT-qPCR |
| FRO6_qRT_R | TCAAGAACATGGTTCCTGTGGTG | RT-qPCR |
| ORE1_qRT_F | ACGTGCCGATGGTACAAAGGTTC | RT-qPCR |
| ORE1_qRT_R | AGGTAAACCGGCTGGTTCCATTC | RT-qPCR |
| SAG13_qRT_F | CTCATATCCTCTGCTGCTGGAGTC | RT-qPCR |
| SAG13_qRT_R | CCAGCTGATTCATGGCTCCTTTG | RT-qPCR |
| SEN4_qRT_F | CATATTGACCGTCGATGACACACC | RT-qPCR |
| SEN4_qRT_R | TCTTTGGAAACAAGACACCGAGAG | RT-qPCR |
| CAB1_qRT_F | TGCACTACTCAACCTCAATGGC | RT-qPCR |
| CAB1_qRT_R | AAAGCTTGACGGCCTTACCG | RT-qPCR |
| CAB4_qRT_F | CCGCTTAACTTTGCTCCTACGC | RT-qPCR |
| CAB4_qRT_R | AACATCGCCAACCTCCCGTTTG | RT-qPCR |
| FT_qRT_F | GCTACAACTGGAACAACCTTTGGC | RT-qPCR |
| FT_qRT_R | ATGAATTCCTGCAGTGGGACTTGG | RT-qPCR |
| SOC1_qRT_F | TTCGCCAGCTCCAATATGCAAG | RT-qPCR |
| SOC1_qRT_R | GTTTGGTGCTGACTCGATCCTTAG | RT-qPCR |
| BIM2_qRT_F | AGTGCCTACTCGCATGAGTTATTG | RT-qPCR |
| BIM2_qRT_R | TTGCTTGCGACAGATCAATGCC | RT-qPCR |
| STZ_qRT_F | TCACAAGGCAAGCCACCGTAAG | RT-qPCR |
| STZ_qRT_R | TTGTCGCCGACGAGGTTGAATG | RT-qPCR |
| SVP_qRT_F | TTGAAACTGGTTTGACGCGTGTG | RT-qPCR |
| SVP_qRT_R | TCTTCCGTTAGTTGCGTTCCTTG | RT-qPCR |

| **Names** | **Sequence (5'-3')** | **Purpose** |
| --- | --- | --- |
| WIP5_qRT_F | ACGGTGTTGGTGAGAGCATTAGG | RT-qPCR |
| WIP5_qRT_R | TGATCTTGCTCGTCCGTGGTTG | RT-qPCR |
| MYC2_qRT_F | AAACCACGTCGAAGCAGAGAGAC | RT-qPCR |
| MYC2_qRT_R | TTGGTACAACCGCTCGTAACGC | RT-qPCR |
| GAPC2_qRT_F | ACCACTGTCCACTCTATCACTGC | RT-qPCR |
| GAPC2_qRT_R | TGAGGGATGGCAACACTTTCCC | RT-qPCR |
| PP2A_qRT_F | TAACGTGGCCAAAATGATGC | RT-qPCR |
| PP2A_qRT_R | GTTCTCCACAACCGCTTGGT | RT-qPCR |
| pFRO6_P1_F_ChIP | CTCAAGGCCAAAGAACATTATGG | ChIP-qPCR |
| pFRO6_P1_R_ChIP | GATTGTAATGGTTGTTGTACCA | ChIP-qPCR |
| pFRO6_P2_F_ChIP | TCGTAGCTAAGTCTAAGTGGTACT | ChIP-qPCR |
| pFRO6_P2_R_ChIP | GTTTCACTGTTAGATTGTTTCTGGT | ChIP-qPCR |
| pFRO6_P3_F_ChIP | ATCTCTCTGCTGGATCTTGAAC | ChIP-qPCR |
| pFRO6_P3_R_ChIP | ATTTCAACCACAATTAAGGGTAAGG | ChIP-qPCR |
| pFRO6_P4_F_ChIP | CAACCTAACGTGACACTCTCTC | ChIP-qPCR |
| pFRO6_P4_R_ChIP | TGATGAGAAGTTGGCTCAGAAG | ChIP-qPCR |
| pFRO6_G1_F_ChIP | TGTCGATCATCGGTTCAAGATAG | ChIP-qPCR |
| pFRO6_G1_R_ChIP | ACTCCGGGTAAGATAGCAATTC | ChIP-qPCR |
| PP2A_qRT_F_ChIP | CGGCTTTCATGATTCCCTCT | ChIP-qPCR |
| PP2A_qRT_R_ChIP | GCCTTAAGCTCCGTTTCCTACTT | ChIP-qPCR |

**Table S2** Expression patterns of Fe transport and/or homeostasis-associated genes that were differentially expressed during age-dependent, dark-induced, and excess Fe-induced senescence.

| **Name** | **TAIR ID** | **log_2_(FC): Age** | **log_2_(FC): Dark** | **log_2_(FC): Fe** |
| --- | --- | --- | --- | --- |
| ***FRO6*** | ***AT5G49730*** | -5.39 | -4.64 | -4.49 |
| *FRO7* | *AT5G49740* | -3.94 | -3.64 | -2.52 |
| *IREG1* | *AT2G38460* | -1.40 | -3.97 | -1.35 |
| *CYB561D* | *AT1G26100* | -2.01 | -3.26 | -1.18 |
| *AT3G61750* | *AT3G61750* | -2.01 | -2.62 | -0.98 |
| *AGO10* | *AT5G43810* | -1.40 | -1.22 | -0.87 |
| *IRT3* | *AT1G60960* | 2.77 | 1.37 | 1.12 |

**Table S3** A list of putative binding targets at the *FRO6* promoter with their DAP-seq peak enrichment signals.

| Position | Peak location | Name | Enrichment signal |
| --- | --- | --- | --- |
| chr5:20204630–20204831 | chr5:20204730 | BAM8 | 10 |
| chr5:20205163–20205364 | chr5:20205263 | BPC1 | 18.8 |
| chr5:20204603–20204804 | chr5:20204703 | SPL9 | 21 |
| chr5:20204624–20204825 | chr5:20204724 | MYB55 | 22 |
| chr5:20205131–20205332 | chr5:20205231 | IDD7 | 23 |
| chr5:20204848–20205049 | chr5:20204948 | GTL1 | 27 |
| chr5:20205152–20205353 | chr5:20205252 | WIP5 | 59 |
| chr5:20205156–20205357 | chr5:20205256 | WIP5 | 82 |
| chr5:20204680–20204881 | chr5:20204780 | SVP | 88.9 |
| chr5:20204669–20204870 | chr5:20204769 | STZ | 116 |
| chr5:20204623–20204824 | chr5:20204723 | BIM2 | 775.5 |
| chr5:20204591–20204792 | chr5:20204691 | BIM2 | 851.5 |
